# Phosphorus-mediated alleviation of aluminum toxicity revealed by the iTRAQ technique in *Citrus grandis* roots

**DOI:** 10.1371/journal.pone.0223516

**Published:** 2019-10-15

**Authors:** Lin-Tong Yang, Yang-Fei Zhou, Yan-Yu Wang, Yan-Mei Wu, Bing Qian, Heng Wang, Li-Song Chen

**Affiliations:** 1 Institute of Plant Nutritional Physiology and Molecular Biology, College of Resources and Environment, Fujian Agriculture and Forestry University, Fuzhou, China; 2 Fujian Provincial Key Laboratory of Soil Environmental Health and Regulation, Fujian Agriculture and Forestry University, Fuzhou, China; Key Laboratory of Horticultural Plant Biology (MOE), CHINA

## Abstract

*Citrus grandis* seedlings were irrigated with nutrient solutions with four Al-P combinations [two Al levels (0 mM and 1.2 mM AlCl_3_·6H_2_O) × two P levels (0 μM and 200 μM KH_2_PO_4_)] for 18 weeks. Al dramatically inhibited the growth of *C*. *grandis* seedlings, as revealed by a decreased dry weight of roots and shoots. Elevating P level could ameliorate the Al-induced growth inhibition and organic acid (malate and citrate) secretion in *C*. *grandis*. Using a comparative proteomic approach revealed by the isobaric tags for relative and absolute quantification (iTRAQ) technique, 318 differentially abundant proteins (DAPs) were successfully identified and quantified in this study. The possible mechanisms underlying P-induced alleviation of Al toxicity in *C*. *grandis* were proposed. Furthermore, some DAPs, such as GLN phosphoribosyl pyrophosphate amidotransferase 2, ATP-dependent caseinolytic (Clp) protease/crotonase family protein, methionine-*S*-oxide reductase B2, ABC transporter I family member 17 and pyridoxal phosphate phosphatase, were reported for the first time to respond to Al stress in *Citrus* plants. Our study provides some proteomic details about the alleviative effects of P on Al toxicity in *C*. *grandis*, however, the exact function of the DAPs identified herein in response to Al tolerance in plants must be further investigated.

## Introduction

Aluminum (Al) is the most abundant metal in the earth’s crust, constituting ~7% of the soil. At neutral pH, Al primarily exists as relatively insoluble aluminosilicate. However, when the soil pH is < 5, rhizotoxic Al^3+^ ions are dissolved into soil solutions from clay minerals, which inhibit crop growth and lead to significant crop yield losses [1]. Al toxicity is a constraining factor that limits crop production and quality in acidic soils, which constitute approximately 40% of the global arable land. The Al-induced growth inhibition or yield loss has been reported in many plants including rice [2, 3], *Arabidopsis* [4], sorghum [1], wheat [5], soybean [6] and *Citrus* [7–10]. Therefore, it is of great importance to understand the physiological and/or biochemical flexibility and underlying molecular mechanism of plant adaptation to Al stress in acidic soil.

The well-documented Al resistant mechanism is the exudation of organic anions (OAs), such as malate, citrate and oxalate, from plant roots. The released OAs can chelate the toxic form of Al into the nonphytotoxic Al-OA complex on the root surface or in the rhizosphere, thus preventing Al from binding to root cell components [11, 12]. By using subtractive hybridization or mapping-based cloning, *ALMT1* and *SbMATE*, which were responsible for malate or citrate secretion, were identified from wheat and sorghum, respectively [13, 14]. Subsequently, more genes or proteins involved in OA exudation or Al-induced signal transduction were isolated and identified in plants, for example, *ALS1* and *AtSTAR1* in *Arabidopsis* [15, 16], *Hm*VALT and *Hm*PALT in hydrangea [17], *HvAACT1* in barley [18], and *NRAT1*/ART1 in rice [19]. With the development of experimental techniques, especially omics-based isolation and identification of molecular compounds, large-scale screening of Al-responsive genes, proteins or metabolites has recently become possible. By using two-dimensional electrophoresis (2-DE) coupled with the Time of Flight (TOF)/TOF-tandem-MS method, Yang et al. [3] found that most DAPs in rice treated with Al were functionally associated with plant hormone-dependent signaling transduction, antioxidant and detoxification. Interestingly, this study reported that sulfur (S) metabolism related enzymes, such as cysteine synthase, played a functional role in Al adaptation for rice. Up-regulation of DAPs, such as S-adenosylmethionine, oxalate oxidase, malate dehydrogenase, cysteine synthase, and ascorbate peroxidase, was also reported in wheat roots by Al treatment [5]. By applying multichannel iTRAQ technology, DAPs involved in the antioxidant system, such as superoxide dismutase (SOD), peroxidase (POD) and catalase (CAT), were found to be upregulated by Al in an Al-tolerant sorghum line, whereas lignification of the root cell wall was not as serious as in the Al-sensitive line [1]. In our previous proteomic study, the higher Al tolerance of *C*. *sinensis* than *C*. *grandis* was attributed to the upregulation of sulfur metabolism, antioxidation, carbohydrate and energy metabolism, cellular transport, jasmonic acid biosynthesis and fine-tuning of protein phosphorylation [20].

Phosphorus (P) is another limiting factor that affects crop growth and production on acid soil. Due to its sorption to soil colloid, incorporation into organic compounds and precipitation with Al or iron, the availability of P in soil was very scarce to plants [21]. Our previous studies indicated that elevating P level could ameliorate Al-induced root OA exudation, translocation of Al from roots to shoots, photosynthesis and growth inhibition in *Citrus* plants [10, 22, 23]. Similar results were also observed in soybean [24], wheat [25], oil tea [26], *Lespedeza bicolor* [27], maize [28] and rice [29]. However, the molecular mechanisms of the alleviative effects of P on Al toxicity in plants have remained unclear.

Citrus plants are ever-green fruit trees cultivated in tropical and subtropical areas and are susceptible to Al toxicity and P deficiency. Previous literature has revealed that some nutrients and chemical compounds can ameliorate Al toxicity in plants, for instance, the alleviative effects of magnesium and salicylic acid on Al toxicity in soybean [30], calcium in *Arabidopsis* [31], silicon in upland rice [32], and sulfur and boron in *C*. *grandis* [7, 8]. To eliminate the harmful effects of Al stress, application of phosphate fertilizer will be an available and valid agricultural procedure. Some details have been studied to investigate the alleviative effects of P on Al toxicity in plants by physiological and biochemical approaches; however, the molecular mechanisms underlying the alleviative effects of P on Al toxicity are not yet understood [10, 23, 24]. In this study, we used an iTRAQ technique to identify and quantify DAPs that might play key roles in the alleviative effects of P on Al-toxicity in *C*. *grandis* roots.

## Material and methods

### Plant material and treatment

This experiment was conducted in Fujian Agriculture and Forestry University, Fuzhou, Fujian, China. *C*. *grandis* seeds were germinated in a plastic tray containing clean river sands and kept moist by spraying with water. After germination, uniform seedlings with three leaves were chosen to plant in 6-L pottery pots (two seedlings per pot) containing clean river sands and supplied with nutrient solution every two days under natural temperature and light in greenhouse. The nutrient solution contained the following macronutrients (in mM): KNO_3_, 1; Ca(NO_3_)_2_, 1; KH_2_PO_4_, 0.2; and MgSO_4_, 0.5; and micronutrients (in μM): H_3_BO_3_, 10; MnCl_2_, 2; ZnSO_4_, 2; CuSO_4_, 0.5; (NH4)_6_Mo_7_O_24_, 0.065; and Fe–EDTA, 20. Seven weeks after transplanting, treatments were initiated and applied for 18 weeks. There were four treatments: 0 (−Al) and 1.2 mM AlCl_3_·6H_2_O (+Al) × 0 and 200 μM KH_2_PO_4_, and each treatment contained 20 pots. The pH value of the nutrient solutions was adjusted to 4.1–4.2 by addition of HCl or NaOH. After treatment, approximately 0.5-mm-long root apices were excised and immediately frozen in liquid nitrogen. Samples were stored at -80°C until extraction.

### Contents and secretion of organic acids in the root apex of *C*. *grandis*

Organic acid secretion in the root apex was collected and measured according to Yang et al. [23]. Approximately 17 weeks after the beginning of P-Al treatments, approximately 5 mm of the root apex was excised from intact roots and washed three times by immersion into 20 ml 0.5 mM CaCl_2_ (pH = 4.1~4.2). The root apices were transferred to 2-mL centrifuge tubes containing 1 mL control solution in the absence or presence of 0.5 mM AlCl_3_·6H_2_O (pH 4.1~4.2). The tubes were placed vertically on a shaker at a rate of 200 rpm in the dark. The duration times for malate and citrate collection were 12 and 24 h, respectively. After the experiment, malate was assayed in a 1 ml reaction mixture containing 50 mM 3-amino-1-propanol–HCl (pH 10), 30 mM glutamate–Na–NaOH (pH 10), 2.7 mM NAD, 1 unit glutamate–oxaloacetate transaminase (GOT, EC 2.6.1.1), 2 mM EDTA, 10 unit NAD–malate dehydrogenase (NAD–MDH, EC 1.1.1.37) and 50 μL exudates. Citrate was measured in a 1 mL reaction mixture containing 100 mM Tris–HCl (pH 7.6), 0.2 mM NADH, 7 unit lactate dehydrogenase (LDH, EC 1.1.1.37), 14 unit NAD–MDH, 0.5 unit citrate lyase (EC 4.1.3.6), 2 mM EDTA and 200 μL exudates. The contents of malate and citrate of *C*. *grandis* roots were measured according to our previous methods [23].

### Measurements of root and shoot dry weight, P and Al contents

At the end of the experiment, ten plants per treatment from different pots (one plant per pot) were harvested and washed thoroughly with deionized water. The plants were divided into roots and shoots and their dry weight measured after being fixed for 30 min at 120°C and oven-dried to achieve a constant weight at 80°C.

After measurement of the dry weight, all samples were ground in a pulverizer and screened through a 100-mesh sieve. Powdered samples of roots and leaves were digested overnight in a mixed solution of nitric acid and perchloric acid (5:1, v/v). The sample solutions were heated, cooled and supplemented with deionized water to a constant volume. P and Al in the solution samples were measured using the ammonium molybdate/ascorbic acid spectrophotometric assay [33] and the aluminon reagent, respectively [34]. There were five replicates for the P and Al measurements.

### Measurements of starch, glucose, H_2_O_2_ production, TBARS, lignin contents, total soluble proteins and enzyme activity

For starch and glucose measurement, about 100 mg root samples were extracted three times with 80% (*v*/*v*) ethanol at 80°C. After the extracts were evaporated, the resulting pellets were thoroughly dissolved with 3 mL ddH_2_O. Glucose was measured in 1 mL reaction mixture containing 100 mM inidazole-HCl (pH 7.9), 5 mM MgCl_2_, 0.5 mM NAD, 1 mM ATP, 2 units G6PDH and 2 units hexokinase. Starch was determined by enzyme kinetic method as glucose equivalents with G6PDH and hexokinase. H_2_O_2_ production was measured according to Yang et al. [7]. The content of H_2_O_2_ was calculated with an extinction coefficient of ε = 26.6 cm^−1^ mM^−1^. TBARS was extracted and measured according to the methods described by Hodges et al. [35]. For lignin measurement, root tip samples were homogenized in 95% ethanol. After centrifugation at 1500 g for 5 min, the pellet was washed three times with 95% ethanol and twice with ethanol-hexane (1:2, v/v). The washed pellet was allowed to air-dry. The lignin content was determined according to the method of Morrison with some modifications [36]. Total soluble proteins were extracted and measured using the method described by Bradford [37]. The enzyme activity of GlPX and PEPC was measured according to methods described by Guo et al. [8] and Yang et al. [23]. There were four replicates for the H_2_O_2_, TBARS, lignin content and enzyme activity assays.

### Protein extraction and mass spectrum analysis

Proteins were extracted from *C*. *grandis* roots according to the method described by Yang et al. [38]. Briefly, approximately 1 g frozen mixed roots from five seedlings (one seedling per pots) were ground into a fine powder in liquid N_2_ with a mortar and pestle. Four milliliters of ice-cold buffer [100 mM Tris-HCl pH 7.8, 100 mM KCl, 50 mM L-ascorbic acid, 1% (v/v) Triton X-100, 1% (v/v) β-mercaptoethanol, and 1 mM phenylmethylsulfonyl fluoride] was added to the powder. The mixture was allowed to thaw slowly on ice and gently pulverized for another two minutes. An equal volume of Tris-saturated phenol (pH 8.0) was added to the resulting homogenate and centrifuged at 13,000 *g* for 15 min at 4°C. The phenolic phase was transferred to a 50-mL Corning tube with five volumes of methanol (containing 100 mM ammonium acetate). The protein pellets were collected and resuspended in 25 mL of ice-cold methanol for 2 h at -20°C after centrifugation at 13,000 g for 15 min at 4°C. Protein pellets were collected by centrifugation at 13,000 g for 15 min at 4°C, washed two times with 25 mL of ice-cooled acetone, and then dried by lyophilization.

### Identification and annotation of DAPs

iTRAQ analysis was conducted by Beijing Genomics Institute (BGI, Shenzhen, China). Phenol-extracted protein samples (100 μg each) from different samples were reduced with 50 mM Tris-(2-carboxyethyl)-phosphine (TCEP), alkylated with 200 mM methyl methane-thiosulfonate (MMTS) in isopropanol, digested using sequencing-grade trypsin (Promega), and labeled using iTRAQ 8-plex kits (AB Sciex Inc., USA) according to the manufacturer’s manual. Here, protein samples of 0P, 0P+Al, 200P, and 200P+Al were labeled with 113, 115, 117 and 119 iTRAQ tags, respectively. After labeling and quenching, the samples were combined and lyophilized before redissolving in 4 mL of 25% (v/v) acetonitrile + 25 mM NaH_2_PO_4_ (pH 3.0) for fractionation by SCX chromatography using a Shimadzu LC-20AB system. Elution was monitored by measuring the absorbance at 214 nm, and fractions were collected every 1 min. The eluted peptides were pooled into 20 fractions, desalted with a Strata X C18 column (Phenomenex) and vacuum-dried. Each fraction was resuspended in buffer A (5% ACN, 0.1%FA) and centrifuged at 20000 g for 10 min. Ten microliters of the supernatant was loaded on a LC-20AD nanoHPLC (Shimadzu, Kyoto, Japan) by the autosampler onto a 2 cm C18 trap column. Data acquisition was performed with a TripleTOF 5600 System (AB SCIEX, Concord, ON) fitted with a Nanospray III source (AB SCIEX, Concord, ON) and a pulled quartz tip as the emitter (New Objectives, Woburn, MA). Protein identification and quantification were performed using MASCOT (version 2.3.02, Matrix Science Inc, Boston, MA). The following search settings were used: peptide tolerance was set at 10 ppm, and MS/MS tolerance was set as 0.05 Da; MS/MS fragment ion mass tolerance of ± 0.1 Da; threshold set-off at 0.05 Da in the ion-score cut-off; tryptic peptides with ≤1 missed cleavage site; pyrophosphorylation of glutamine and variable oxidation of methionine and iTRAQ labeling of tyrosine were set as variable modifications; carbamidomethylation of cysteine and iTRAQ labeling of lysine and the N-terminal amino group of peptides were set as fixed modifications. Peptide change was set as Mr, and monoisotopic masses were chosen. The iTRAQ 8plex was chosen for quantification during the search simultaneously. The search results were passed through additional filters before exporting the data. For protein identification, the filters were set as follows: significance threshold *p* < 0.05 (with 95% confidence) and an ion score or expected cutoff less than 0.05 (with 95% confidence). For protein quantitation, the filters were set as follows: “median” for protein ratio type; minimum precursor charge set to 2+ and minimum peptides set to 2. Median intensities were set as normalization, and outliers were removed automatically. The peptide threshold was set as above for Identity. Searches were performed against the *C*. *clementina* database (JGI version 0.9, www.phytozome.org/clementine, 35976 sequences). Proteins with ratios with *p* <0.05 and fold changes >1.5 were considered DAPs.

### Bioinformatic analysis of proteins

DAPs were mapped to Gene Ontology Terms by online blast analysis against a reference database on the website (http://www.geneontology.org/). According to the biological processes that were mapped, the DAPs were classified into several categories, including carbohydrate and energy metabolism, stress response, cell wall and cytoskeleton, antioxidation and detoxification, protein metabolism, nucleic acid metabolism, cell transport, signal transduction, lipid metabolism and unknown proteins.

### Quantitative RT-PCR (qRT-PCR) analysis of selected gene expression

Genes of 15 DAPs were selected to perform qRT-PCR to complement the results of iTRAQ. Total RNA was isolated from 0P, 0P+Al, 200P, and 200P+Al root samples using TRIzol reagent (Invitrogen, Carlsbad, CA). First-strand cDNA was synthesized using the RevertAid^TM^ First-Strand cDNA Synthesis Kit (Thermoscientific) following the manufacturer’s instructions. The consequent cDNA was diluted to 200 μL using TE buffer. Gene-specific primers were designed using Primer Premier (Version 5.0) according to the corresponding sequences deposited in the *C*. *clementina* database. The sequences of the forward and reverse primers used are listed in Table 1. qRT-PCR was performed in 20 μL of reaction mixture containing 10 μL Bestar^™^ qPCR Master Mix SYBR Green (DBI Bioscience, Shanghai, China), 0.4 μL of 10 μM forward primer, 0.4 μL of 10 μM reverse primer, 2 μL cDNA template and 7.2 μL ddH_2_O using a CFX96 Touch^™^ Deep Well Real-Time PCR Detection System (Bio-Rad, Hercules, CA, USA). Samples for qRT-PCR were run in five biological replicates with two technical replicates. Relative gene expression was calculated using the ddCt algorithm. For normalization of gene expression, the polyubiquitin and U4/U6 small nuclear ribonucleoprotein gene were selected as internal standards, and samples from *C*. *grandis* treated with 0P were used as the reference sample, which was set to 1.

**Table 1:** Special gene primer pairs for qRT-PCR of DAPs.

Accession number	Protein name	Forward primer	Reverse primer
Ciclev10009763m|PACid:20793057	Ribosomal protein S11-beta (RPS11)	ATGAACAGAACCATCATCGTTAGGCG	CCACCAGAAGACCCAGCAGGTATCA
Ciclev10022435m|PACid:20810058	Ribosomal protein L18e/L15 superfamily protein (RPL18)	TCACCGATGACATTAGGGCTTACGA	CCTTCCTCTAGCACGCTCAAACTTCC
Ciclev10019935m|PACid:20809278	UDP-glucose 6-dehydrogenase family protein (UDPGD)	GCCAAAAGGCGATTCAAGCACTG	GCAAGAACCACCAAAACCAACACTG
Ciclev10028714m|PACid:20814721	Isocitrate dehydrogenase 1 (ICDH1)	CTTGCGGATGGGTTATTCTTGGAA	GCTGATGAAAGAAGCAAAGCCACTG
Ciclev10031821m|PACid:20805442	Glyceraldehyde-3-phosphate dehydrogenase-like family protein (G3PDH)	GTCCACGCCGTATTCGGTATGAGT	GGTTCAGTGTATCCCAGGCTTTTAGC
Ciclev10020878m|PACid:20811851	Phosphoenolpyruvate carboxylase family protein (PEPC)	TCCCTATGATTGACTCCCCAGAAGC	GCACTCAAATCCAACGGTCCCAT
Ciclev10004268m|PACid:20791548	Beta galactosidase 9 (GAL9)	GAGGCAGAATGGACTGATTTGACACG	TCGGAATTGTAGGCTCCACGGTAAT
Ciclev10017635m|PACid:20816899	Glycosyl hydrolase family 38 protein (GH38)	ATTTGGTTCCTCACTCCCACGAT	TGCCTTACTTTGCTGCCTCCA
Ciclev10000256m|PACid:20786659	Starch branching enzyme 2.2 (SBE2.2)	AAGTACCCTCGACCTCCAAAACCC	CCACCAAGACCCGCAGACCTAA
Ciclev10027732m|PACid:20814761	Pre-mRNA-processing protein 40B (mRPP)	CACTGCCTCTGCTCCGCTACCTAC	TGGGCTTGATACTATCACTTCAACCGT
Ciclev10021867m|PACid:20807674	Tonoplast intrinsic protein 2;3 (TIP2)	CCGCTGAAGGTTTGGTTATGGAAA	TCCTCAGTAGAGGCTGGGGTGTAAGA
Ciclev10009186m|PACid:20794673	ABC transporter I family member 17(ABCT17)	GAGTTGAGAAAAGAATCGGATGATGGG	AAGAGGAGTCGAGGTCTGCAAGTGAG
Ciclev10009304m|PACid:20794954	Glutathione peroxidase 7 (GPX7)	TCGTTCCTTTGGTGTTCACGCTACA	CAGGGTTTGATCCAGGCTCTTGC
Ciclev10012473m|PACid:20797152	aluminum sensitive 3 (ALS3)	GTGCTGCTGGCTGTCCTGTTGTC	CATTCCTGCGACTGGGATGATGTA
Ciclev10014212m|PACid:20816315	lipoxygenase 2 (LOX2)	ATTACCGTATGTGGGGAAACTGAGACA	CGTCCAAAGCCAGGAGTCAGTAGAA
Ciclev10017283m|PACid:20816634	Polyubiquitin	CCAACGATCAATCGGCTCACATC	AATCTCATCACCATCTTCCATTTCCAG
Ciclev10031363m|PACid:20805012	U4/U6 small nuclear ribonucleprotein	ACTCATGGGAACGGCTGGTGGTC	TCGGCAGGCACGCATCCTTAGAG

### Principal component analysis (PCA) of DAPs

The abundances of all the DAPs from *C*. *grandis* roots treated with different P-Al combinations were normalized and transformed for PCA using the *princomp* function in R circumstance (Version 3.4.3). The PCA loading plot generated by Sigmaplot 10.0 was used to visualize two loadings against each other to investigate the relationships between the variables.

### Experimental design and statistical analysis

Experiments were conducted with 4–10 replicates. The results are displayed as the means ± SE (n = 4–10). Differences among the four treatment combinations were analyzed by two (P levels) × two (Al levels) ANOVA. Means were separated by Duncan's new multiple range test at *p* <0.05.

## Results

### Plant biomass, P and Al contents in response to different P-Al treatments

**Fig 1:**
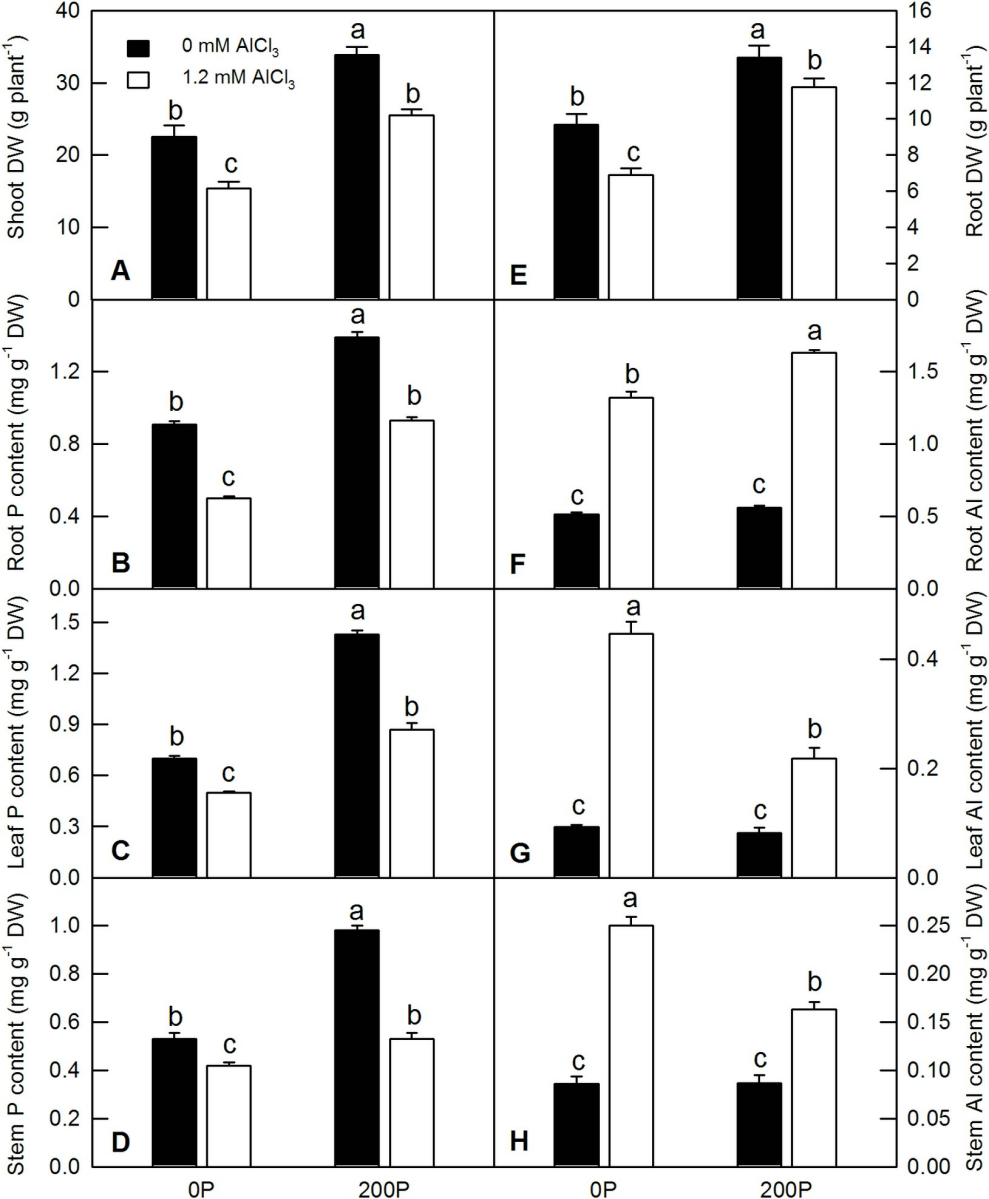
**Effects of P-Al interaction on plant dry weight (DW; shoot, A; root, E), P content (root, B; leaf, C; stem, D) and Al content (root, F; leaf, G; stem, H) in *C*. *grandis* seedlings.** Bars represent means ± SE (n = 5 for P and Al contents or 10 for plant DW). Differences among the four treatment combinations were analyzed by 2 (B levels) × 2 (Al levels) ANOVA. Different letters indicate a significant difference at *p* < 0.05.

Both low P and Al treatments dramatically decreased the dry weight of shoots and roots in *C*. *grandis* (Fig 1A and 1E). Elevating the P supply could significantly increase the dry weight of shoots and roots under Al treatment (Fig 1A and 1E), indicating that the higher P could ameliorate the inhibitory effects of Al in *C*. *grandis*. The shoot dry weight was reduced by 31.5% in response to Al treatment under a low P level, whereas it was reduced by 24.5% under a high P level (S1A Fig). The root dry weight was reduced by 28.9% in response to Al treatment under a low P level, whereas it was reduced by 12.1% under a high P level (S1A Fig). Under the–Al condition, the shoot dry weight was reduced by 33.4% in response to a low P compared to 39.6% under the +Al condition (S1B Fig). Both a low P level and Al treatment significantly decreased the P content in roots, leaves and stems (Fig 1B, 1C and 1D). Without Al treatment, there was no significant difference between the Al contents of roots, leaves and stems under different P levels (Fig 1F, 1G and 1H). Al treatment significantly increased the Al content of roots, leaves and stems in *C*. *grandis* at any given P level. Under Al treatment, elevating the P supply increased the root Al content, whereas it decreased the leaf and stem Al contents (Fig 1F, 1G and 1H).

### H_2_O_2_ production and the contents of starch, glucose and TBARS in *C*. *grandis* roots under different P-Al treatments

**Fig 2:**
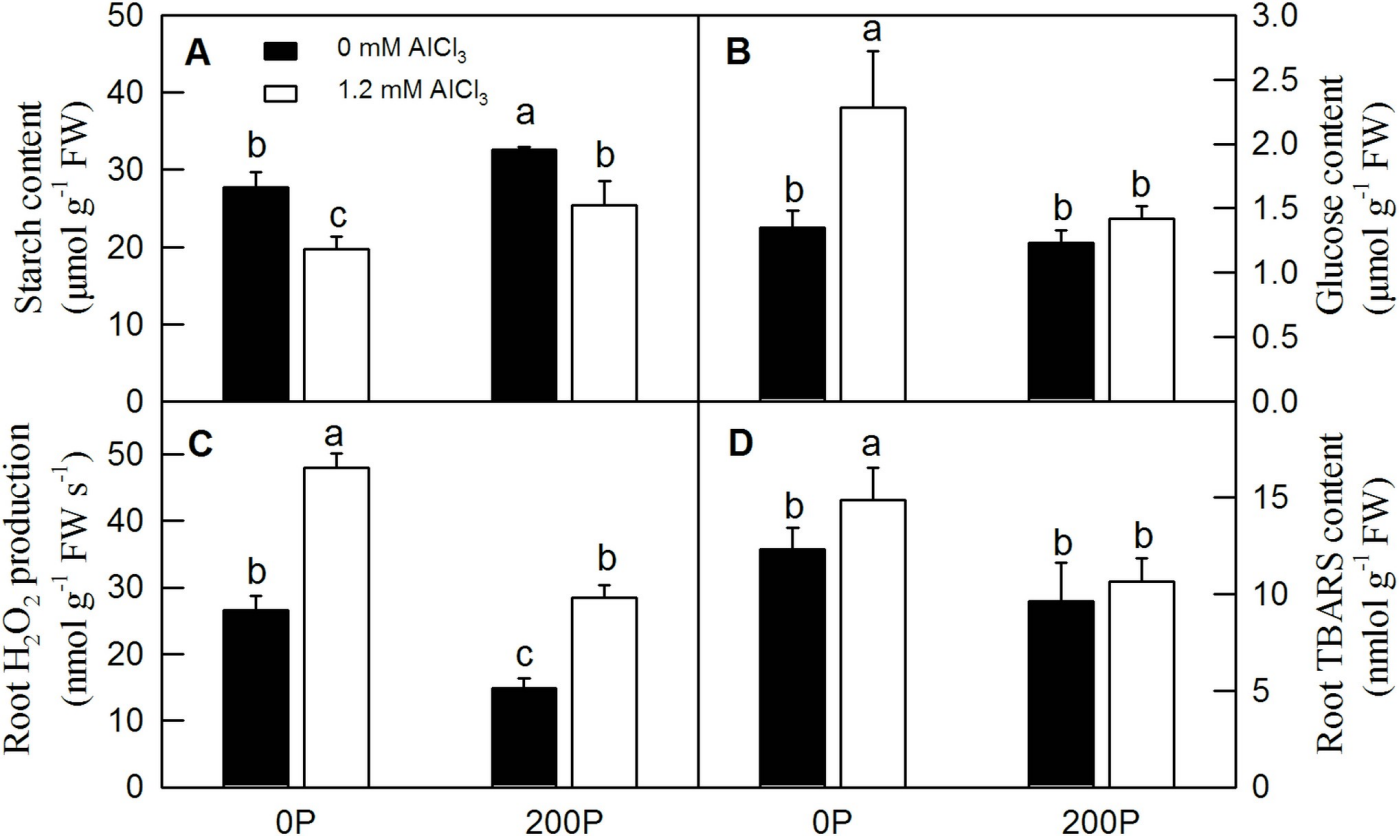
**Effects of the P-Al interaction on root H**_**2**_**O**_**2**_ **production (C) and the contents of starch (A), glucose (B) and TBARS (D) in *C*. *grandis* seedlings.** Bars represent means ± SE (n = 4). Differences among the four treatment combinations were analyzed by 2 (P levels) × 2 (Al levels) ANOVA. Different letters indicate a significant difference at *p* < 0.05.

**Fig 3:**
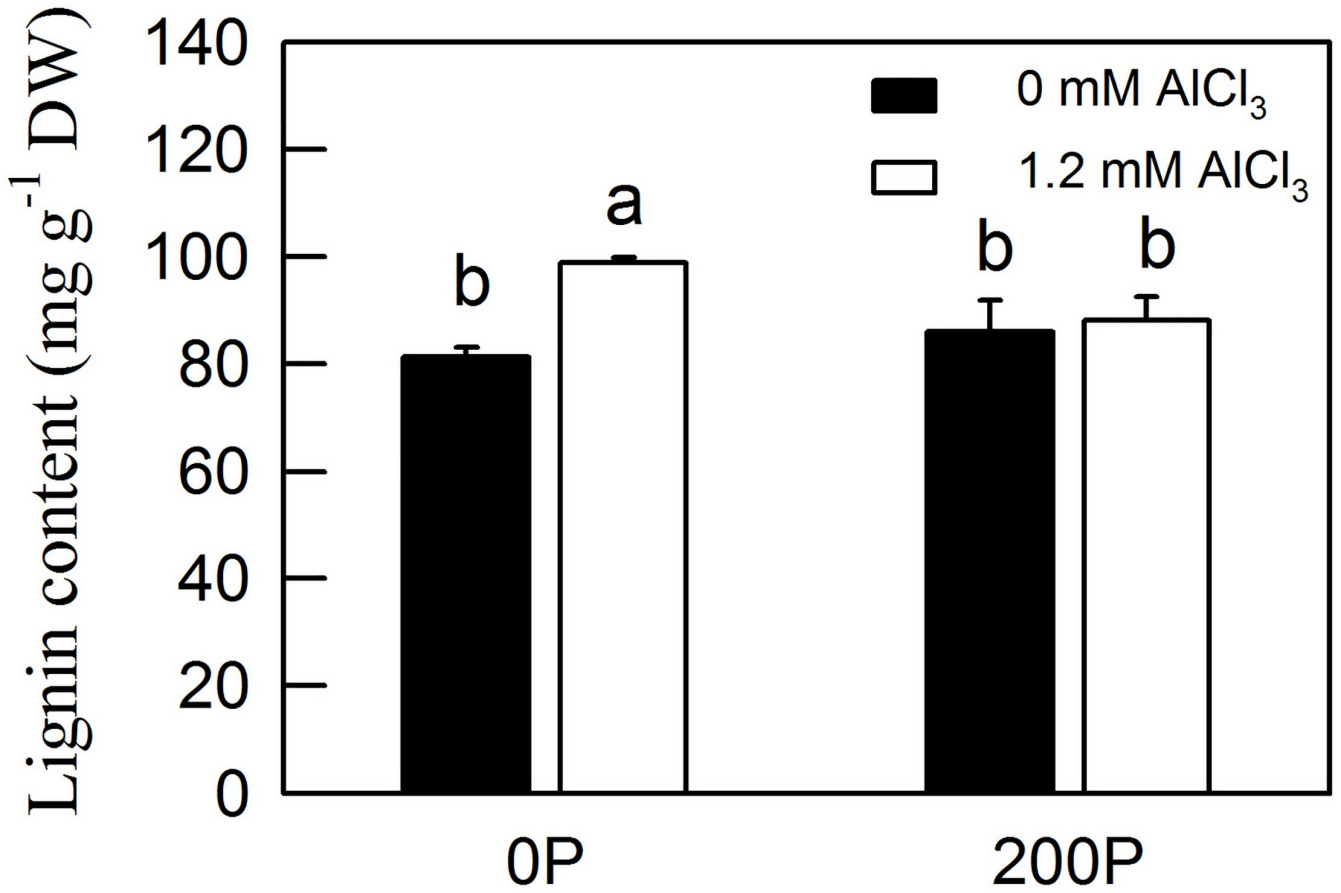
Lignin content of *C*. *grandis* roots under different P-Al treatments. Bars represent means ± SE (n = 4). Differences among the four treatment combinations were analyzed by 2 (P levels) × 2 (Al levels) ANOVA. Different letters indicate a significant difference at *p* < 0.05.

The starch content and H_2_O_2_ production was significantly increased by both Al and low P in *C*. *grandis* roots (Fig 2A and 2C). The glucose content was significantly increased by Al only under low P level (Fig 2B). The TBARS and lignin contents were dramatically increased by Al treatment in *C*. *grandis* roots under a low P level, but they were similar between 200P-Al and 200P+Al. Both H_2_O_2_ production and TBARS content were significantly elevated in 0P+Al compared with 200P+Al roots (Figs 2C, 2D and 3).

### Enzyme activities of phosphoenolpyruvate carboxylase (PEPC) and glutathione peroxidase (GlPX), contents and secretion of malate and citrate in *C*. *grandis* roots under different P-Al treatments

**Fig 4:**
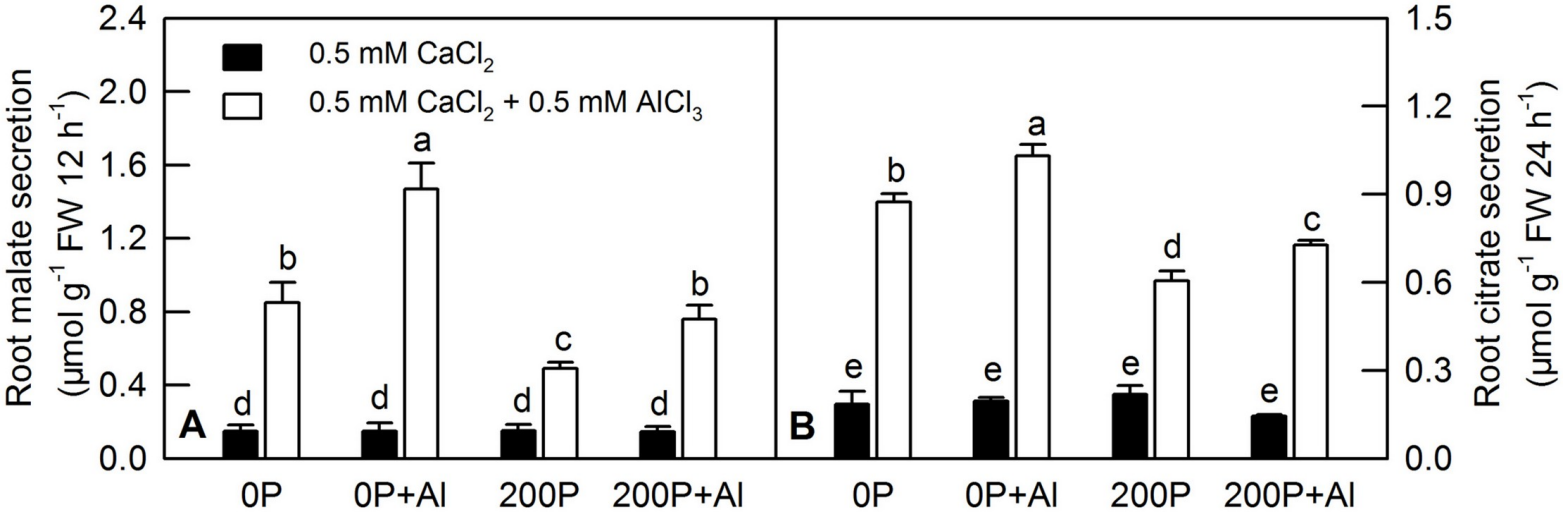
Al-induced secretion of malate and citrate in *C*. *grandis* roots. Malate (A) and citrate (B) secretion from excised roots was measured after 12 or 24 h treatment, respectively, in 0.5 mM CaCl_2_ + 0.5 mM AlCl_3_·6H_2_O or 0.5 mM CaCl_2_ solution, pH 4.1–4.2. Bars represent means ± SE (n = 4). Differences among the eight treatments were analyzed by 4 (pretreatments) × 2 (Al levels) ANOVA. Different letters indicate a significant difference at *p* < 0.05.

**Fig 5:**
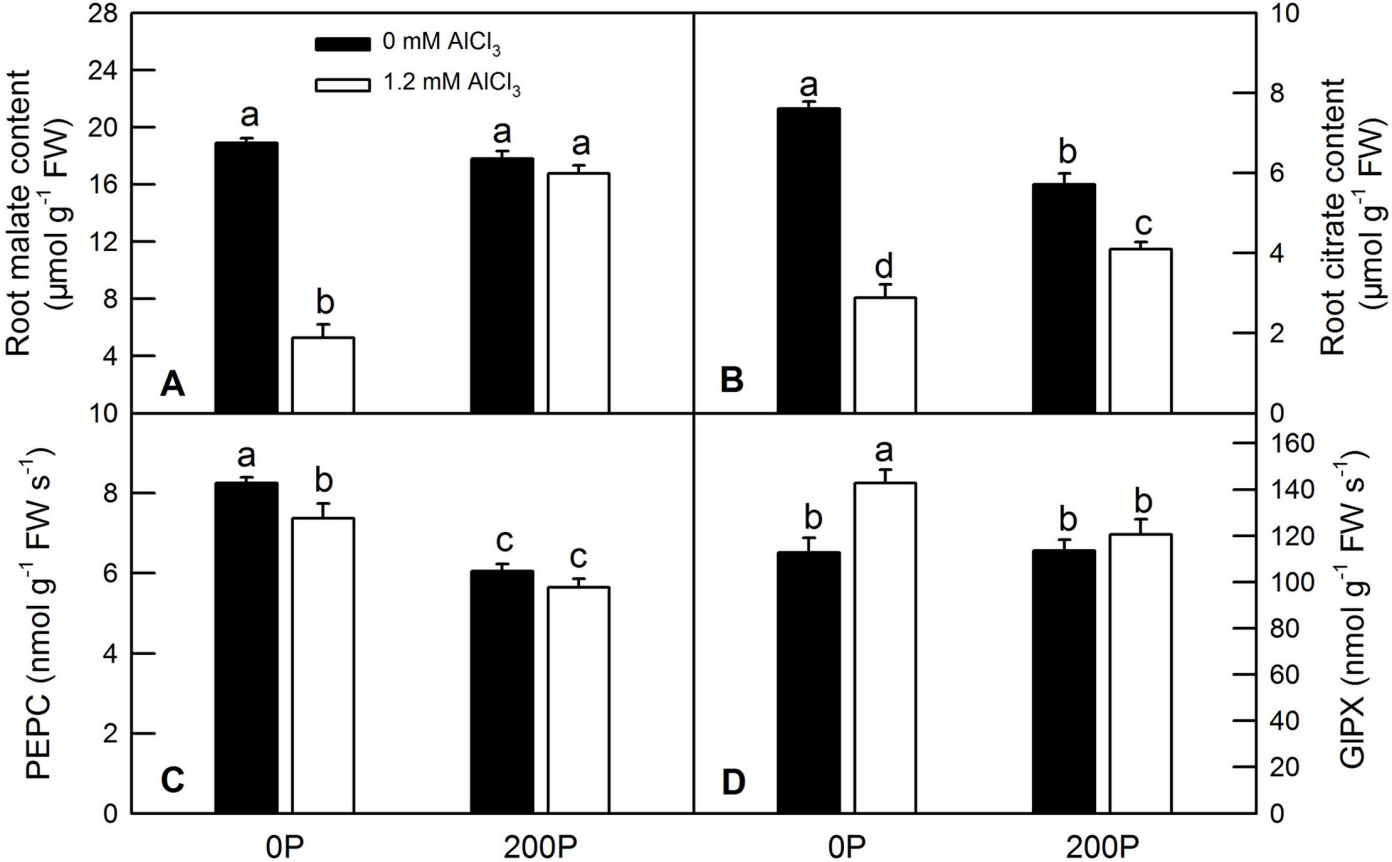
**Contents of malate (A) and citrate (B) and activity of two enzymes (PEPC, C; GlPX, D) in *C*. *grandis* roots.** Bars represent means ± SE (n = 4). Differences among the four treatment combinations were analyzed by 2 (P levels) × 2 (Al levels) ANOVA. Different letters indicate a significant difference at *p* < 0.05.

Al incubation induced higher secretion of malate and citrate in *C*. *grandis* roots compared with the control solution (0.5 mM CaCl_2_). A low P supply increased the secretion of malate and citrate regardless of whether the seedlings had received Al-preculture (Fig 4). Al treatment greatly increased the secretion of malate and citrate compared with the control ones, whereas elevating the P supply decreased Al-induced malate and citrate secretion (Fig 4A and 4B). Al dramatically decreased the contents of malate and citrate in *C*. *grandis* roots under a low P level. Under a high P condition, Al did not change the content of malate, but it decreased the content of citrate (Fig 5A and 5B). Under a low P condition, Al treatment decreased or increased the enzyme activities of PEPC and GlPX, respectively, but it did not change either of them under a high P level (Fig 5C and 5D).

### iTRAQ analysis, DAP annotation and principal component analysis (PCA) loading plot

Our iTRAQ analysis generated a total of 412595 spectra, 48979 unique spectra and 20288 unique peptides in *C*. *grandis* roots. The lengths of most peptides identified in this study (92.18% in proportion) ranged from 7 to 20 amino acids. A protein search were performed against the *C*. *clementina* database and resulted in the identification of a total of 4876 proteins. According to the criteria of the fold change >1.5 and *P* value < 0.05, a total of 318 DAPs were identified, of which 311 DAPs were induced by Al treatment under a low P level, whereas only 46 DAPs were induced by a high P level in *C*. *grandis* roots. Based on the GO annotation result, the 318 DAPs were clustered into nine catalogues: protein metabolism (74 DAPs), carbohydrate and energy metabolism (73 DAPs), nuclear acid metabolism (14 DAPs), cellular transport (21 DAPs), stress response (46 DAPs), lipid metabolism (19 DAPs), biological regulation and signal transduction (18 DAPs), cell wall and cytoskeleton metabolism (29 DAPs), other biological process (54 DAPs) (Table 2).

**Table 2:** Summary of DAPs in different biological processes.

Biological processes	0P vs 0P+Al		200P vs 200P+Al	
	Down-regulated	Up-regulated	Down-regulated	Up-regulated
Protein metabolism	39	32	12	2
Carbohydrate and energy metabolism	8	47	2	6
Cellular transport	8	12	0	1
Nuclear acid metabolism	4	6	1	1
Stress response	7	40	1	8
Lipid metabolism	3	11	2	2
Biological regulation and signal transduction	5	14	1	0
Cell wall and cytoskeleton metabolism	9	17	1	1
Other biological process	13	36	1	4
Total	96	215	21	25

**Fig 6:**
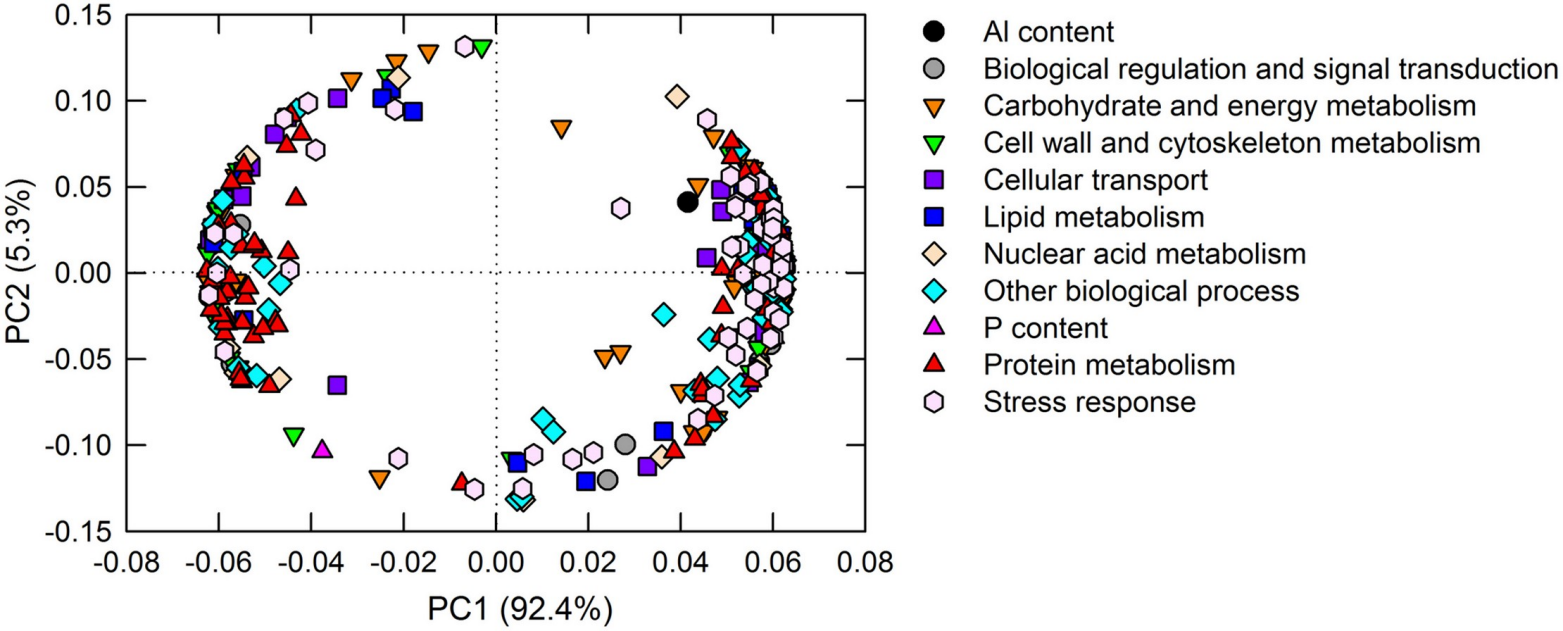
Principal component analysis (PCA) loading plot of the DAPs in roots under different P-Al treatments. Three hundred eighteen DAPs from *C*. *grandis* roots were transformed for PCA analysis. The first two PCs explained 97.7% of the biological variation in response to P-Al treatments, with PC1 accounting for 92.4% and PC2 5.3%.

PCA of the 318 DAPs was carried out and presented in Fig 6. The first two PCs explained 97.7% of the biological variation in response to four P-Al combinations with PC1 accounting for 92.4% and PC2 accounting for 5.3%. Interestingly, the Al content in roots was highly clustered with DAPs involved in the stress response.

### qRT-PCR analysis of DAP genes in response to P-Al treatments

**Fig 7:**
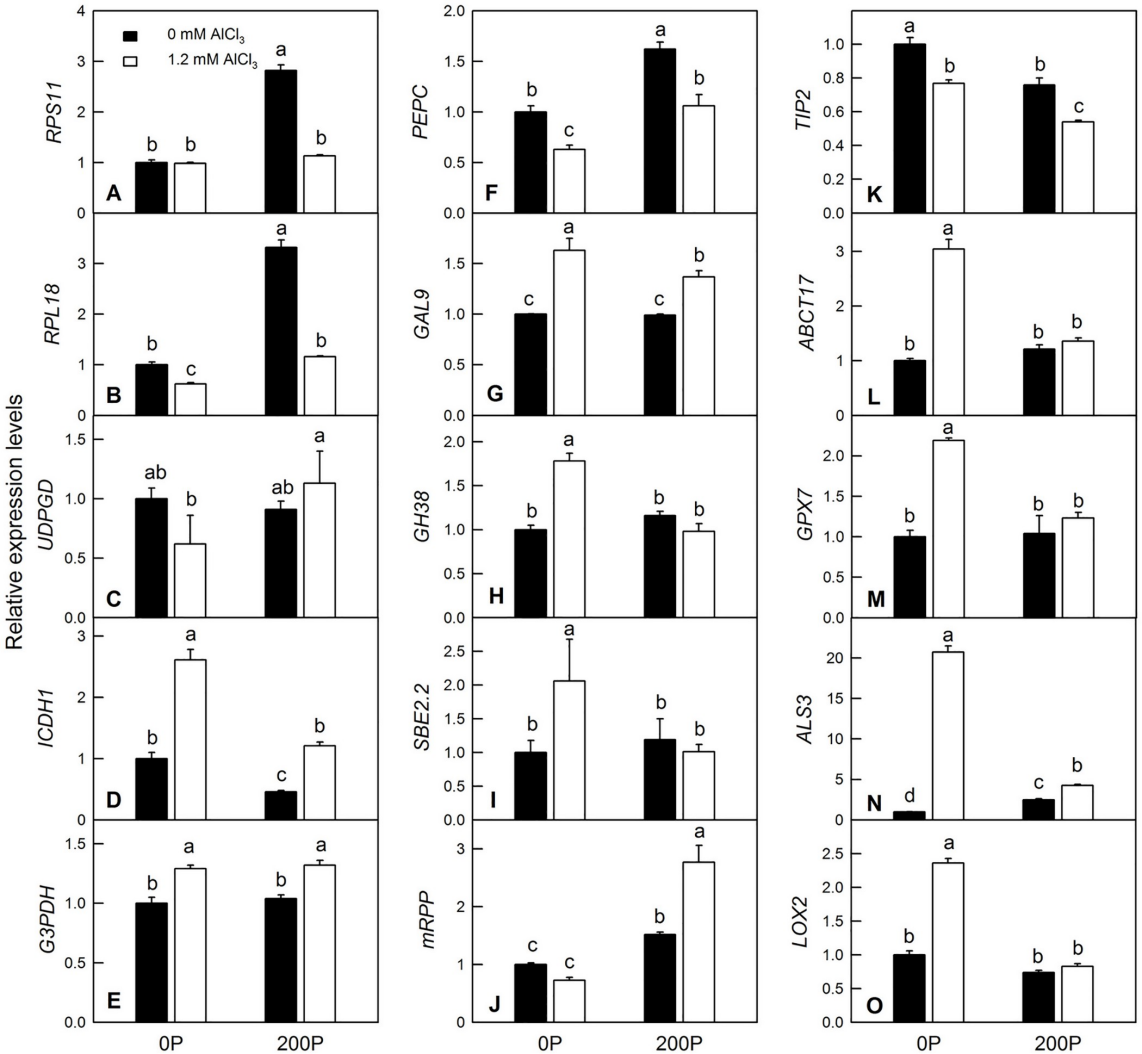
Relative expression levels of genes encoding 15 DAPs under P-Al treatments. A: Ribosomal protein S11-beta (RPS11); B: ribosomal protein L18e/L15 superfamily protein (RPL18); C: UDP-glucose 6-dehydrogenase family protein (UDPGD); D: isocitrate dehydrogenase 1 (ICDH1); E: glyceraldehyde-3-phosphate dehydrogenase-like family protein (G3PDH); F: phosphoenolpyruvate carboxylase family protein (PEPC); G: beta-galactosidase 9 (GAL9); H: glycosyl hydrolase family 38 protein (GH38); I: starch branching enzyme 2.2 (SBE2.2); J: pre-mRNA-processing protein 40B (mRPP); K: tonoplast intrinsic protein 2;3 (TIP2); L: ABC transporter I family member 17(ABCT17); M: glutathione peroxidase 7 (GPX7); N: aluminum sensitive 3 (ALS3); O: lipoxygenase 2 (LOX2). Bars represent means ± SE (n = 5). Differences among the four treatment combinations were analyzed by 2 (P levels) × 2 (Al levels) ANOVA. Different letters indicate a significant difference at *p* < 0.05.

To complement the iTRAQ results, special primer pairs of 15 DAPs genes were designed and synthesized. qRT-PCR analysis revealed that the expression patterns of *RPL18* involved in protein metabolism, *UPDGD*, *ICDH1*, *G3PDH*, *GAL9*, *GH38* and *SBE2*.*2* involved in carbohydrate and energy metabolism, *ABCT17* involved in cellular transport, *GPX7* and *ALS3* involved in the stress response and *LOX2* involved in lipid metabolism were in accordance with the proteomic data in response to Al treatment under 0 μM P. Likewise, the expression patterns of *RPS11*, *UPDGD*, *ICDH1*, *GH38*, *SBE2*.*2*, *GPX7*, *ALS3* and *LOX2* were consistent with the proteomic data under 200 μM P (Fig 7). These results indicated that most of the selected DAPs were regulated at the transcriptional level, and the iTRAQ technique was a reliable method to quantify DAPs in *C*. *grandis* roots. Discrepancies between the transcriptional patterns and proteomic observations of some proteins might be caused by post-translational modifications (PTMs) such as phosphorylation and ubiquitination [39].

## Discussion

### P alleviates Al-induced growth inhibition in *C*. *grandis*

Al toxicity and P deficiency are two important constraining factors that limited the production and quality of agricultural products in acidic soil. Previous literatures have revealed that some nutrients and chemical compounds can ameliorate Al toxicity in plants, such as the alleviative effects of magnesium on Al toxicity in soybean (*Glycine max*), rice bean (*Vigna umbellata*) and broad bean (*Vicia faba*) [40], calcium in *Arabidopsis* [31], silicon in upland rice [32], sulfur in *C*. *grandis* [8] and salicylic acid [30]. The beneficial effects of those divalent cations or chemical compounds are mostly related to their role in increasing the capacity of ion uptake, suppressing Al uptake, restoring root cell membrane integrity and membrane selective permeability, and reducing reactive oxygen species (ROS) levels and ROS-induced oxidative damage. The beneficial effects of P in plant Al tolerance have been observed in many plants, including *Citrus* [10, 23, 24]. Here, we showed that Al treatment dramatically reduced the dry weight of roots and shoots of *C*. *grandis*, whereas elevating P level in nutrient solution could effectively ameliorate the Al-induced growth inhibition (Fig 1). Both low P and Al treatment significantly decreased the P content of roots, leaves and stems in *C*. *grandis* (Fig 1B, 1C and 1D). Despite increasing the Al content of roots, elevating P level significantly decreased the Al content in leaves and stems in *C*. *grandis* under Al treatment (Fig 1F, 1G and 1H). These results are consistent with previous studies in *C*. *sinensis* [23], soybean [24], oil tea [26] and *Lespedeza bicolor* [27], which also found that elevating P level or preculture with P could confer Al tolerance in the abovementioned plants. According to element measurements, the intuitive mechanism of P-alleviated Al toxicity has been mostly attributed to the chemical precipitation of Al by P in the zone of P incorporation. It is less persuasive to declare that the unique role of P in alleviating Al toxicity in plants is due the chemical precipitation of Al, even though elevating the P supply could actually restrict most of the absorbed Al in the root zone and inhibit the transport of Al to shoots, as revealed in the current study (Fig 1).

### DAPs involved in protein metabolism in response to the P-Al interaction

Disordered protein metabolism has been observed in previous studies investigating abiotic stress in plants, including Mg deficiency in *C*. *sinensis* [20, 41], boron deficiency or excess in *C*. *sinensis* and *C*. *grandis* [38, 42], iron deficiency and salinity stress in *Arabidopsis* [43, 44], and Al stress in sorghum [1], rice [2, 3], *Arabidopsis* [4], wheat [5] and soybean [6]. Here, we found that 39 downregulated and 32 upregulated DAPs involved in protein metabolism were induced by Al treatment under the low P level, whereas only 12 downregulated and two upregulated DAPs were observed under the high P level (Table 2). Interestingly, most of the downregulated DAPs were ribosomal protein family members, which are involved in protein translation, and most of the upregulated DAPs were proteinases or protein kinases (such as eukaryotic aspartyl protease, amidase family protein, CLP protease proteolytic subunit 1 and dual specificity protein phosphatase), which are mainly involved in protein degradation or PTMs. Measurement of total soluble proteins in *C*. *grandis* roots revealed that Al treatment significantly decreased the total soluble proteins in *C*. *grandis* roots under the low P level, whereas no change was observed under a high P level (S2 Fig). The alleviation of Al-induced inhibition of protein translation and degradation or PTM of proteins by beneficial nutrients has also been reported in *C*. *grandis* roots during B-Al interactions [7]. Using the iTRAQ technique, Wang et al. [2] found that the Al-sensitive rice cultivar had more DAPs and higher relative abundance of DAPs related to protein metabolism than the Al-tolerant one under Al stress and suggested that Al stress disturbed the equilibrium of protein metabolism more strongly in the Al-sensitive than in the Al-tolerant cultivar.

Glutamate can participate in the TCA cycle by cytosolic transamination of glutamate and conversion to 2-oxyglutarate inside the mitochondrial matrix. Here, we reported that some DAPs, such as glutamate dehydrogenase and glutamate decarboxylase, which participated in glutamate metabolism, were induced in *C*. *grandis* roots by Al treatment under a low but not under a high P level, indicating that *C*. *grandis* roots suffered more severe Al toxicity under a low than under a high P level. Up-regulation of DAPs related to glutamate metabolism has also been observed in rice under Al stress and in *Arabidopsis* under salinity stress [44, 45]. Furthermore, some DAPs, such as the subtilase family protein, GLN phosphoribosyl pyrophosphate amidotransferase 2, ATP-dependent caseinolytic (Clp) protease/crotonase family protein, papain family protein and methionine-*S*-oxide reductase B2, are reported for the first time to be responsive to Al stress in *Citrus* plants. Further research is necessary to investigate the exact role of these proteins in the Al response in plants.

### DAPs involved in carbohydrate and energy metabolism

Carbohydrate and energy metabolism are the basis of plant growth, development and morphogenesis. The alternation of carbohydrate and energy metabolism by abiotic stress has been observed in many studies. For instance, Al stress significantly increased the protein abundance of aconitase, malic enzyme and pyruvate decarboxylase in the Al-sensitive rice cultivar ‘Kasalath’, whereas only slight changes in the abundances of these enzymes were observed in the Al-tolerant cultivar ‘Koshihikari’ [2]. Our previous studies reported that both low P and Al stress could alter the differential expression of genes and proteins related to the glycolysis pathway and tricarboxylic acid (TCA) cycle in *Citrus* roots [20, 22]. Our present proteomic results showed that ten glycosyl hydrolases (S1 Table, #333, #2060, #916, *etc*.), five UDP-glucosyltransferases (S1 Table, #1520, #883, #1764, *etc*.) and two beta galactosidases (S1 Table, #2735, #3305; Fig 7G) were upregulated by Al treatment under low P condition, but remained constant or slightly decreased under high P condition. Wang et al. [45] reported that sucrose biosynthesis of different Al-tolerant rice varieties increased with prolongation of the Al treatment time, indicating that upregulated sucrose metabolism plays an important role in the Al toxicity response in plants. The alleviative effects of P might be partially due to the vital role of high P in maintaining stable sucrose metabolism in *C*. *grandis* roots under Al treatment as revealed by lower change variation of starch and glucose contents caused by Al under high P than low P condition (Fig 2A and 2B). Triosephosphate isomerase (TPI) and glyceraldehyde-3-phosphate dehydrogenase (GAPDH) are two key enzymes in the glycolysis pathway. Al significantly decreased and increased the protein abundance of TPI (S1 Table, #1380) and GAPDH (S1 Table, #2428; Fig 7E) under low P condition, respectively, but there was no significant difference between treatment and control roots under high P condition, which indicated that an adequate P supply could ensure normal energy metabolism in *C*. *grandis* roots (S1 Table). Excluding the disturbance of glycolysis, the metabolism of starch was also impaired by Al treatment under low P condition. For example, both beta-amylase 6 (#2760) that catalyzes the hydrolysis of starch into sugars and glucose-1-phosphate adenylyltransferase (#1617) involved in the biosynthesis of starch were upregulated by Al treatment under low P but remained constant under high P condition. The upregulation of energy-related proteins such as ferredoxin–nitrite reductase, glucose-6-phosphate isomerase and fructose-1, 6-bisphosphatase, phosphoglycerate kinase, malate dehydrogenase, pyruvate dehydrogenase, aldolase, alpha-ketoglutarate dehydrogenase, and aconitate hydratase, among others, has also been observed in rice, soybean or *Arabidopsis* under Al treatment [46, 47]. Moreover, the protein abundance of pyridoxal phosphate phosphatase involved in the biogenesis of vitamin B6 was upregulated by Al treatment both under low and high P conditions, indicating that induction of vitamin B6 metabolism might be an adaptive strategy [48].

Secretion of organic acids in the root apex is an important mechanism of Al tolerance in plants. Here, we found that both low P and Al treatment could induce the secretion of malate and citrate in *C*. *grandis* roots. Elevating P level could ameliorate Al-induced secretion of malate and citrate (Fig 4). However, such secretion of organic acids was not positively correlated with internal contents of malate and citrate (Fig 5A and 5B), consistent with our previous study [23]. PEPC catalyzes the carboxylation of phosphoenolpyruvate (PEP) to yield oxaloacetate (OAA) and inorganic phosphate, which replenish intermediates of the citric acid cycle and provide metabolites for nitrogen assimilation and amino acid synthesis [49]. Here, we found that two PEPC isoenzymes (S1 Table, #2036 and #3019) were downregulated by Al treatment under low P level, whereas no change was observed under high P level. The downregulated protein abundance of PEPC was consistent with the qRT-PCR (Fig 7F) and our enzymatic assay (Fig 5C) results [50]. The downregulated PEPC might be due to a lower P content in *C*. *grandis* roots induced by Al, because low P level has also been reported to reduce PEPC activity in some C4 species such as maize and *Cynodon dactylon* [51].

### DAPs involved in cellular transport, cell wall and cytoskeleton metabolism

The well-known mechanism of Al tolerance in both monocots and dicots is the release of organic acid anions from roots into the rhizosphere in response to Al. These anions are able to chelate Al to form the nonphytotoxic Al form [52]. Other metabolites such as UDP-glucose or its derivatives are released from vesicles into the apoplast and used to modify cell walls to mask the sites for Al binding, elevating Al tolerance in plants [7, 15]. Thus, the concert of cellular transport is crucial for Al resistance in plants. Previous studies have revealed that Al can upregulate several membrane transporters at the gene or protein level, such as ALMTs, MATEs, VALT and PALT [13, 14, 17]. Here, we showed that Al treatment downregulated eight proteins involved in cellular transport, such as voltage-dependent anion channel 4, nuclear transport factor 2, plasma membrane intrinsic protein 1B and ARF-GAP containing protein, whereas it upregulated 12 proteins, including annexin 4, ABC1 family protein, tonoplast monosaccharide transporter2, tonoplast intrinsic protein 2;3, sucrose transporter 4, and ABC transporter I family member 17, under low P condition (S1 Table, #400; Fig 7L). However, Al did not alter the abundance of these proteins other than upregulating the ABC transporter I family member 17 under high P condition, indicating that *C*. *grandis* roots suffered more severe Al toxicity under low P than under high P condition (S1 Table, #400). Furthermore, the simultaneous downregulation and upregulation of RAN GTPase (S1 Table, #984 and #27) and SNARE-like superfamily protein (S1 Table, #1372 and #1183) by Al under low P condition indicated that Al might impair cargo transport mediated by those two proteins.

Alteration of root cell wall and cytoskeleton components under Al treatment has been observed in many plants, including sorghum, *Arabidopsis*, rice and *Citrus* [1, 7, 53]. A previous study shown that Al-induced ROS accumulation resulted in hyperaccumulation of lignin content in sorghum roots, leading to root cell wall lignification and a reduction of root cell elongation [1]. Here, we found that the induction of some enzymes by Al under low P condition, such as laccase 7 and *O*-methyltransferase, could increase the content of lignin in *C*. *grandis* roots, which was verified by measurement of H_2_O_2_ production and lignin content (Fig 3). The higher H_2_O_2_ production and lignin content in *C*. *grandis* roots under low P compared with high P further demonstrated that Al treatment could alter re-modeling of the secondary cell wall in *C*. *grandis* roots and that adequate P could protect Al-induced lignification and elongation of root cells (Figs 2C and 3). Furthermore, the downregulation of several tubulin isozymes and pectin lyase-like superfamily protein by Al in *C*. *grandis* roots under low P coincided with the reduction of cell replication and elongation, as revealed by the decrease of root dry weight (S1 Table, #570, #486, #2600 and #1784; Fig 1E).

### DAPs involved in the stress response

Al toxicity can trigger lipid peroxidation and ROS production in plant roots [54]. To cope with the increased oxidative stress, upregulation of the antioxidant system, including antioxidant enzymes and detoxification metabolites, has been observed in rice [47], trifoliate orange [55], tobacco [56] and maize [57]. S metabolism plays a crucial role in the detoxification of ROS in plants. Our previous studies have shown that increasing S can upregulate its absorption and increase *S*-containing compounds and related metabolic enzymes in *Citrus* roots exposed to Al stress [8]. Here, we found that seven glutathione *S*-transferase family proteins (S1 Table, #2322, #29 and #1136, *etc*.), one glutathione peroxidase (S1 Table, #2174; Figs 5D and 7M), one *S*-adenosylmethionine carrier (S1 Table, #647) and one *S*-adenosyl-L-methionine-dependent methyltransferase superfamily protein (S1 Table, #2145) were significantly upregulated by Al under low P, while no difference was observed under high P condition. This finding is in agreement with our previous report, which demonstrated that S metabolism was upregulated both in *Citrus* roots and leaves [20, 58]. Glycosyl hydrolase is considered to play various functions in cell wall metabolism, plant defense, signal transduction and starch hydrolysis [59]. Here, we found that Al upregulated ten glycosyl hydrolase family proteins under low P, but it did not change their protein abundances under high P condition (S1 Table; Fig 7H). This result indicated that elevating P level could ameliorate Al-induced oxidative stress. The dynamic change in glycosyl hydrolases was also observed in B-induced alleviation of Al toxicity in *C*. *grandis* roots [7]. Moreover, stress-related proteins such as nudix hydrolase homolog 20, ferritin 4, pathogenesis-related thaumatin superfamily protein, IAA-leucine-resistant-like 3, P-glycoprotein 11, osmotin 34, *Arabidopsis* defensin-like protein and aluminum sensitive 3 were also significantly induced by Al under low P but not high P (S1 Table).

### DAPs involved in lipid metabolism and nuclear acid metabolism

Lipids are one of the major components of biological membranes and play important roles in plant responses to abiotic stress. Abiotic stresses, such as water deficit and temperature stress, can trigger lipid-dependent signaling cascades, which activate plant adaptation processes [60]. The lipid composition of root cells, as well as the total and relative contents of the various phospholipid classes, is known to be markedly altered under a variety of stress conditions [61]. Here, we found that Al downregulated and upregulated three and 11 proteins involved in lipid metabolism under low P condition, respectively (S1 Table). Except for calcium-dependent lipid-binding family proteins (S1 Table, #2781), Al did not change any of their protein abundances under high P condition. Among these DAPs, the upregulation of four lipoxygenases (LOX) was consistent with our previous report, which showed that both the gene expression level and protein abundance of LOX were upregulated by Al and concluding that both jasmonic acid biosynthesis and levels might be elevated in response to Al in *Citrus* roots (S1 Table, #1525, #1113, #1055 and #1778; Fig 7O) [9, 20]. HXXXD/BAHD acyl-transferases catalyze acyl transfer reactions between CoA-activated hydroxycinnamic acid derivatives and hydroxylated aliphatics and play key roles in extracellular lipid biosynthesis to preserve water and provide protection in adverse conditions [62]. The upregulation of the HXXXD-type acyl-transferase family proteins in *C*. *grandis* roots by Al under low P condition might be an adaptive strategy as Al treatment could actually decrease the relative water content (RWC) of *Citrus* roots [8].

Al could impair DNA replication, integrity and the cell cycle profile in plant roots [63]. Therefore, nuclear acid metabolism might be altered by Al in *C*. *grandis* roots. Here, we found that Al downregulated DAPs such as arginine/serine-rich splicing factor (SC35), pre-mRNA-processing protein 40B, dsDNA-binding family protein and RNA binding plectin/S10 domain-containing protein, involved in mRNA splicing or nuclear acid processing, and upregulated several proteins, including ribonuclease, which was related to nuclear acid degradation, under low P condition (S1 Table). However, the protein abundance of those DAPs, excluding SC35, was not altered by Al under high P condition (S1 Table), which might indicate that high P protects *C*. *grandis* roots against Al-induced disorders of nuclear metabolism.

### DAPs involved in biological regulation and signal transduction

As sessile organisms, plants must cope with abiotic stress such as nutrient disorders, water deficit and an abnormal temperature. Flexible biological regulation and signaling transduction are necessary to ensure the survival and normal growth of plants [64]. Our proteomic results showed that Al treatment downregulated the protein abundance of calreticulin-1, coatomer epsilon subunit, calcium-binding EF hand family protein, peroxin 11c and peroxin 22 only under low P condition (S1 Table). This result is consistent with previous research showing that long-term B deletion or Al treatment decreases the abundance of Ca-binding and/or calmodulin-related proteins and calreticulin-1 in *C*. *sinensis* [38], *C*. *grandis* [7] and rice [45], respectively. Furthermore, under low P level, Al significantly upregulated the protein abundance of spindle pole body component 98, major facilitator superfamily protein, chromatin-remodeling protein 11, Auxin-responsive GH3, ABA receptor 3, embryo-specific protein 3 (ATS3), germin-like protein 10, and Hercules receptor kinase 2, among others, in *C*. *grandis* roots (S1 Table). This result is consistent with a study showing that Al upregulates the abundance of mitogen-activated protein kinase 1/5, auxin-responsive protein IAA1 and ethylene-responsive transcription factor 1, among others, in rice [47]. Excluding auxin and ethylene responsive proteins, HERCULES receptor kinase, which is involved in brassinosteroid (BR)-mediated growth regulation and developmental processes such as cell elongation, senescence, vascular development and various stress responses, was also upregulated by Al under low P condition (S1 Table, #1849) [65]. The Al-induced increase in OXO (germin) was correlated with root growth inhibition, Al uptake, and cell damage in young barley roots [66]. The upregulation of germin-like protein 10 by Al under low P condition might also be responsible for the increased Al content and decreased biomass of *C*. *grandi*s roots (Fig 1E and 1F).

## Conclusions

Al dramatically inhibited the growth of roots and shoots in *C*. *grandis* seedlings, as revealed by a decreased dry weight of roots and shoots and increased H_2_O_2_ and TBARS contents. P alleviated the Al-induced growth inhibition and organic acid (malate and citrate) secretion in *C*. *grandis*. Using the iTRAQ technique, 318 DAPs were successfully identified and quantified in response to different P-Al combinations. The possible mechanism underlying P-induced alleviation of Al toxicity in *C*. *grandis* may include the following aspects: 1) enhanced the degradation of dysfunctional proteins (proteasome or protease) and deceleration of protein biosynthesis; 2) assurance of the balance of glycolysis and starch biosynthesis; 3) reduced H_2_O_2_ production and protection against Al-induced lignification and elongation of root cells; and 4) enhance mRNA splicing and nuclear acid processing. Furthermore, some DAPs, such as GLN phosphoribosyl pyrophosphate amidotransferase 2, ATP-dependent caseinolytic (Clp) protease/crotonase family protein, methionine-*S*-oxide reductase B2 ABC transporter I family member 17 and pyridoxal phosphate phosphatase were reported to be responsive to Al stress in *Citrus* plants at first time. Overall, our study provides some proteomic details about the alleviative effect of P on Al toxicity in *C*. *grandis*; however, the function of DAPs identified herein needs to be further investigated.

### Data access

The mass spectrometry proteomics data have been deposited to the ProteomeXchange Consortium via the PRIDE partner repository with the dataset identifier PXD012534.

## Supporting information

S1 Fig Reductions of dry-weight of *C*. *grandis* shoots and roots induced by Al and low P treatment. (TIF)

S2 Fig Effects of P-Al interaction on total soluble proteins of *C*. *grandis* roots. Bars represent means ± SE (n = 5). Differences among the four treatment combinations were analyzed by 2 (P levels) × 2 (Al levels) ANOVA. Different letters indicate a significant difference at *p* < 0.05.
(TIF)

S1 Table Differentially abundant proteins (DAPs) identified by using iTRAQ technique under P-Al interaction in *C*. *grandis* roots. (DOC)

S2 Table Raw data for Fig 1 and S1 Fig. (XLSX)

S3 Table Raw data for Fig 2. (XLS)

S4 Table Raw data for Fig 3. (XLSX)

S5 Table Raw data for Fig 4. (XLS)

S6 Table Raw data for Fig 5. (XLSX)

S7 Table Raw data for Fig 6. (XLSX)

S8 Table Raw data for Fig 7. (XLSX)

S9 Table Raw data for S2 Fig. (XLSX)
