# Supplementary figures and images for: Phosphorus-mediated alleviation of aluminum toxicity revealed by the iTRAQ technique in Citrus grandis roots
Source: PLoS One. 2019 Oct 15;14(10):e0223516. doi: 10.1371/journal.pone.0223516 (PMC6793874; doi:10.1371/journal.pone.0223516)

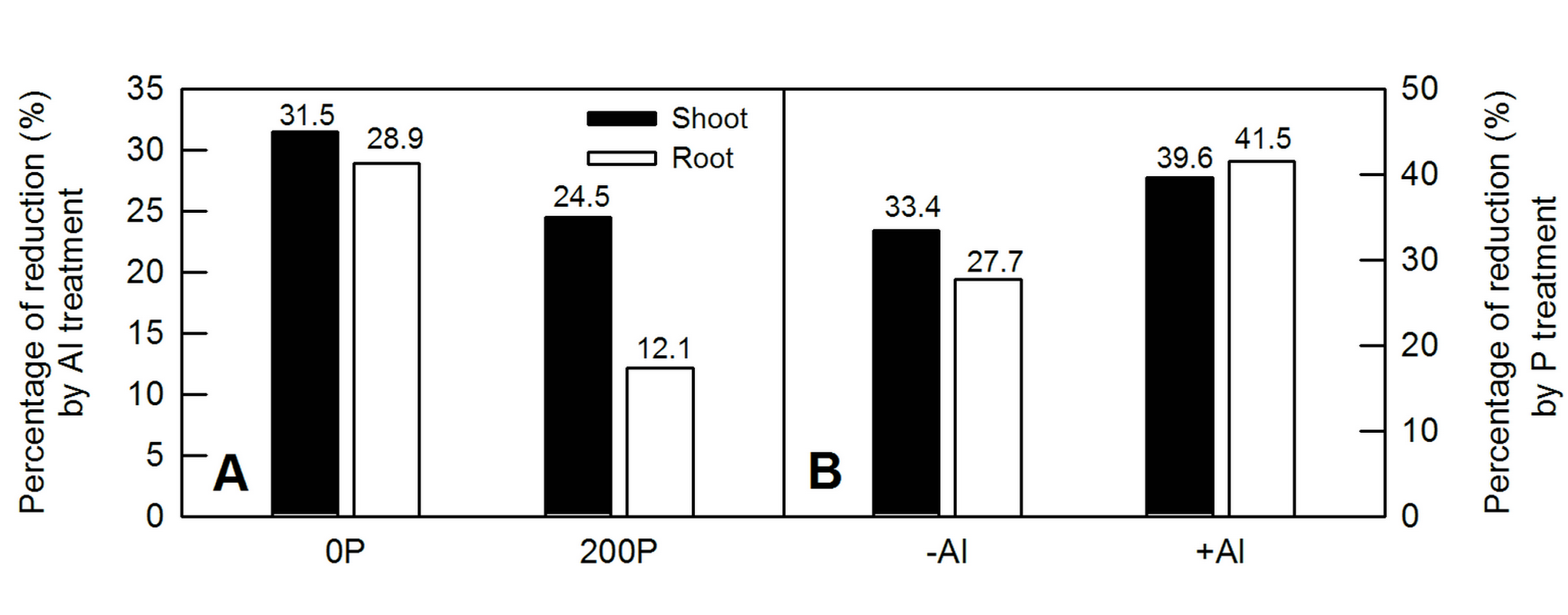

Supplement: S1 Fig — (TIF) [file pone.0223516.s001.TIF]

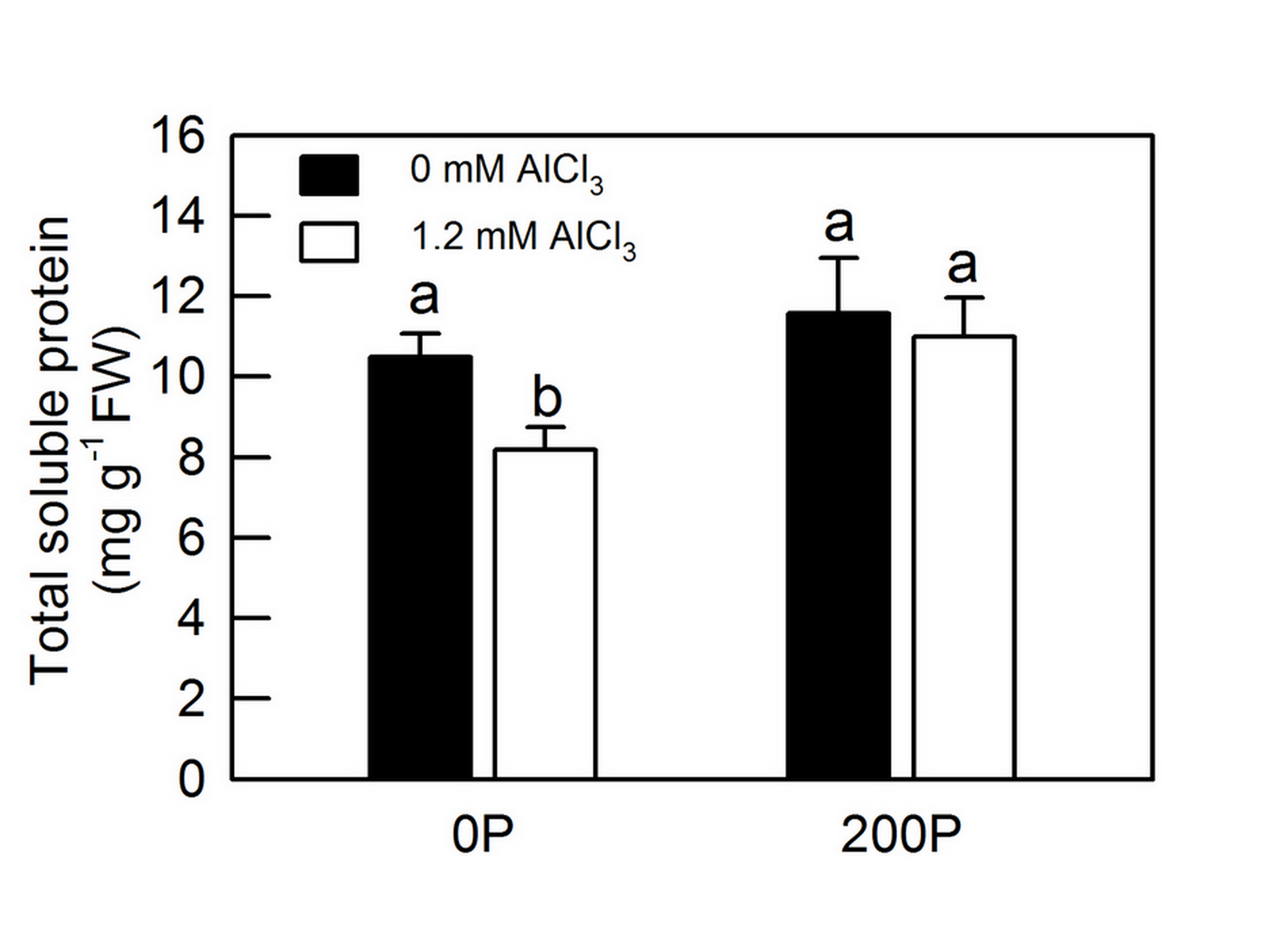

Supplement: S2 Fig — Bars represent means ± SE (n = 5). Differences among the four treatment combinations were analyzed by 2 (P levels) × 2 (Al levels) ANOVA. Different letters indicate a significant difference at p < 0.05. (TIF) [file pone.0223516.s002.tif]
