# Supplementary material for: Phosphorus-mediated alleviation of aluminum toxicity revealed by the iTRAQ technique in Citrus grandis roots
Source: PLoS One. 2019 Oct 15;14(10):e0223516. doi: 10.1371/journal.pone.0223516 (PMC6793874; doi:10.1371/journal.pone.0223516)
Supplement: S1 Table — (DOC) [file pone.0223516.s003.doc]

| **S1 Table.** Differentially abundant proteins (DAPs) identified by using iTRAQ technique under P-Al interaction in *C. grandis* roots | | | | | | | | | | | | | |
| --- | --- | --- | --- | --- | --- | --- | --- | --- | --- | --- | --- | --- | --- |
| **Hits** | **Accession** | **Mass** | **Score** | **Coverage** | **Spectrum** | **Unique Spectrum** | **Peptide** | **Unique Peptide** | **Fold change (Ratio to 0P)** | | | **Homologue in *Arabidopsis*** | **Protein name** |
|  |  |  |  |  |  |  |  |  | **0P vs 0P+Al** | **0P vs 200P** | **0P vs 200P+Al** |  |  |
| **Protein metabolism** | |  |  |  |  |  |  |  |  |  |  |  |  |
| 2478 | Ciclev10007181m|PACid:20790305 | 50742 | 413 | 27.6 | 27 | 3 | 9 | 3 | 0.451 | 0.523 | 0.917 | AT1G03220.1 | Eukaryotic aspartyl protease family protein |
| 3313 | Ciclev10009763m|PACid:20793057 | 24687 | 73 | 11.3 | 5 | 5 | 3 | 3 | 0.338 | 0.805 | 0.628 | AT5G23740.1 | **Ribosomal protein S11-beta** |
| 937 | Ciclev10023466m|PACid:20809809 | 54918 | 254 | 12 | 18 | 6 | 5 | 3 | 0.38 | 0.798 | 0.499 | AT5G48760.1 | Ribosomal protein L13 family protein |
| 927 | Ciclev10033083m|PACid:20804008 | 21344 | 129 | 22.8 | 8 | 8 | 3 | 3 | 0.406 | 0.872 | 0.304 | AT5G02610.1 | Ribosomal L29 family protein |
| 498 | Ciclev10023969m|PACid:20810966 | 42963 | 233 | 12.1 | 8 | 8 | 3 | 3 | 0.407 | 0.88 | 0.556 | AT2G36620.1 | Ribosomal protein L24 |
| 1018 | Ciclev10033116m|PACid:20803807 | 17252 | 91 | 30.4 | 6 | 3 | 3 | 2 | 0.418 | 1.063 | 0.688 | AT1G07070.1 | Ribosomal protein L35Ae family protein |
| 743 | Ciclev10013051m|PACid:20799039 | 18310 | 441 | 61.1 | 13 | 7 | 5 | 3 | 0.441 | 0.723 | 0.884 | AT3G44590.1 | Ribosomal protein family 60S acidic |
| 2217 | Ciclev10020582m|PACid:20810676 | 54202 | 583 | 23 | 21 | 19 | 6 | 5 | 0.482 | 1.105 | 0.567 | AT3G25520.1 | Ribosomal protein L5 |
| 1359 | Ciclev10009798m|PACid:20796123 | 23777 | 211 | 31.3 | 10 | 10 | 4 | 4 | 0.497 | 0.839 | 0.516 | AT2G19730.1 | Ribosomal L28e protein family |
| 1337 | Ciclev10013057m|PACid:20796908 | 22794 | 144 | 15.5 | 9 | 9 | 2 | 2 | 0.504 | 0.733 | 0.529 | AT5G02960.1 | Ribosomal protein S12/S23 family protein |
| 1642 | Ciclev10032800m|PACid:20804334 | 27712 | 261 | 32.5 | 24 | 24 | 9 | 9 | 0.509 | 0.939 | 0.536 | AT5G39850.1 | Ribosomal protein S4 |
| 714 | Ciclev10029426m|PACid:20813110 | 27739 | 249 | 28.2 | 11 | 4 | 4 | 1 | 0.528 | 0.943 | 0.695 | AT3G55280.1 | Ribosomal protein L23AB |
| 650 | Ciclev10013164m|PACid:20798847 | 17210 | 61 | 14.3 | 5 | 2 | 2 | 1 | 0.541 | 0.91 | 0.593 | AT1G07070.1 | Ribosomal protein L35Ae family protein |
| 2739 | Ciclev10026719m|PACid:20802119 | 22605 | 219 | 13 | 13 | 13 | 2 | 2 | 0.542 | 0.746 | 0.492 | AT3G49910.1 | Translation protein SH3-like family protein |
| 2486 | Ciclev10016241m|PACid:20815669 | 35217 | 193 | 32.6 | 12 | 12 | 5 | 5 | 0.548 | 0.685 | 0.863 | AT5G40580.1 | 20S proteasome beta subunit PBB2 |
| 165 | Ciclev10026360m|PACid:20800032 | 39072 | 432 | 25.4 | 25 | 14 | 7 | 5 | 0.552 | 1.08 | 0.82 | AT3G13580.1 | Ribosomal protein L30/L7 family protein |
| 2247 | Ciclev10032494m|PACid:20803323 | 42911 | 492 | 31.7 | 41 | 41 | 8 | 8 | 0.555 | 0.751 | 0.537 | AT3G62870.1 | Ribosomal protein L7Ae/L30e/S12e/Gadd45 family protein |
| 838 | Ciclev10006195m|PACid:20791339 | 21822 | 632 | 29.9 | 38 | 13 | 5 | 3 | 0.563 | 0.842 | 0.779 | AT4G27090.1 | Ribosomal protein L14 |
| 2505 | Ciclev10022435m|PACid:20810058 | 28380 | 419 | 24.6 | 13 | 2 | 4 | 1 | 0.565 | 0.923 | 0.683 | AT5G27850.1 | **Ribosomal protein L18e/L15 superfamily protein** |
| 999 | Ciclev10029626m|PACid:20814707 | 17737 | 351 | 30.6 | 13 | 13 | 4 | 4 | 0.567 | 0.752 | 0.546 | AT4G39200.1 | Ribosomal protein S25 family protein |
| 1069 | Ciclev10032980m|PACid:20804586 | 26843 | 283 | 30.1 | 14 | 7 | 4 | 1 | 0.578 | 0.926 | 0.705 | AT3G55280.1 | Ribosomal protein L23AB |
| 1092 | Ciclev10031073m|PACid:20805254 | 80394 | 90 | 6.2 | 12 | 7 | 4 | 2 | 0.59 | 0.914 | 0.718 | AT1G27150.1 | Tetratricopeptide repeat (TPR)-like superfamily protein |
| 2410 | Ciclev10001267m|PACid:20787865 | 51696 | 135 | 12 | 7 | 6 | 4 | 3 | 0.619 | 0.809 | 0.87 | AT5G64430.1 | Octicosapeptide/Phox/Bem1p family protein |
| 60 | Ciclev10032636m|PACid:20805408 | 38002 | 570 | 28.3 | 27 | 24 | 6 | 5 | 0.619 | 0.998 | 0.822 | AT1G74050.1 | Ribosomal protein L6 family protein |
| 3223 | Ciclev10005301m|PACid:20791981 | 45208 | 103 | 7.1 | 3 | 3 | 2 | 2 | 0.621 | 0.795 | 0.731 | AT5G53140.1 | Protein phosphatase 2C family protein |
| 1634 | Ciclev10013058m|PACid:20798796 | 20144 | 697 | 32.6 | 42 | 42 | 6 | 6 | 0.622 | 0.851 | 0.677 | AT5G04800.1 | Ribosomal S17 family protein |
| 507 | Ciclev10006145m|PACid:20790323 | 23543 | 467 | 48.3 | 29 | 29 | 9 | 9 | 0.63 | 0.93 | 0.563 | AT4G00100.1 | Ribosomal protein S13A |
| 1662 | Ciclev10005021m|PACid:20792098 | 53992 | 221 | 9.6 | 11 | 11 | 3 | 3 | 0.632 | 0.937 | 0.614 | AT4G25500.1 | Arginine/serine-rich splicing factor 35 |
| 1374 | Ciclev10022870m|PACid:20811532 | 19878 | 524 | 36 | 23 | 12 | 4 | 3 | 0.648 | 0.788 | 1.069 | AT3G05560.1 | Ribosomal L22e protein family |
| 760 | Ciclev10002830m|PACid:20789294 | 17782 | 160 | 19.7 | 14 | 14 | 3 | 3 | 0.653 | 0.76 | 0.837 | AT1G20580.1 | Small nuclear ribonucleoprotein family protein |
| 2753 | Ciclev10012508m|PACid:20798906 | 39677 | 1055 | 43.3 | 34 | 28 | 9 | 8 | 0.653 | 0.793 | 0.542 | AT4G34670.1 | Ribosomal protein S3Ae |
| 418 | Ciclev10005711m|PACid:20790626 | 38971 | 337 | 23.2 | 15 | 15 | 5 | 5 | 0.654 | 0.872 | 0.655 | AT5G10360.1 | Ribosomal protein S6e |
| 2876 | Ciclev10001453m|PACid:20788226 | 61086 | 367 | 18.8 | 20 | 7 | 7 | 3 | 0.654 | 0.901 | 0.753 | AT1G61580.1 | Ribosomal-protein L3 B |
| 1048 | Ciclev10009312m|PACid:20796106 | 38916 | 428 | 31.3 | 22 | 11 | 7 | 5 | 0.659 | 1.055 | 0.859 | AT3G13580.1 | Ribosomal protein L30/L7 family protein |
| 2889 | Ciclev10002735m|PACid:20785886 | 23789 | 157 | 14.8 | 7 | 7 | 2 | 2 | 0.66 | 0.619 | 0.636 | AT2G40510.1 | Ribosomal protein S26e family protein |
| 2655 | Ciclev10012856m|PACid:20796870 | 29151 | 1008 | 49.2 | 34 | 34 | 11 | 11 | 0.661 | 0.902 | 0.896 | AT5G16130.1 | Ribosomal protein S7e family protein |
| 2818 | Ciclev10004309m|PACid:20791116 | 113291 | 5137 | 49.8 | 200 | 76 | 33 | 14 | 0.662 | 0.93 | 0.829 | AT1G56070.1 | Ribosomal protein S5/Elongation factor G/III/V family protein |
| 2180 | Ciclev10019417m|PACid:20808545 | 74108 | 737 | 23.9 | 27 | 15 | 10 | 5 | 0.664 | 0.729 | 0.77 | AT3G25800.1 | Protein phosphatase 2A subunit A |
| 2288 | Ciclev10004661m|PACid:20791414 | 75484 | 894 | 34.5 | 40 | 5 | 15 | 2 | 0.665 | 0.646 | 0.846 | AT5G26360.1 | TCP-1/cpn60 chaperonin family protein |
| 1344 | Ciclev10005000m|PACid:20791333 | 54786 | 61 | 12.6 | 6 | 5 | 5 | 4 | 1.067 | 2.094 | 0.789 | AT1G03220.1 | Eukaryotic aspartyl protease family protein |
| 768 | Ciclev10011157m|PACid:20798143 | 104083 | 97 | 7.9 | 5 | 3 | 5 | 3 | 1.501 | 1.271 | 1.393 | AT1G69670.1 | Cullin 3B |
| 1490 | Ciclev10025395m|PACid:20801007 | 66485 | 158 | 7.2 | 5 | 5 | 3 | 3 | 1.503 | 0.931 | 1.036 | AT1G79720.1 | Eukaryotic aspartyl protease family protein |
| 208 | Ciclev10000363m|PACid:20787324 | 92373 | 82 | 6.7 | 5 | 5 | 4 | 4 | 1.503 | 0.987 | 1.135 | AT5G67360.1 | Subtilase family protein |
| 715 | Ciclev10020136m|PACid:20807277 | 55129 | 108 | 7.6 | 3 | 3 | 2 | 2 | 1.511 | 1.488 | 1.196 | AT1G64660.1 | Methionine gamma-lyase |
| 2791 | Ciclev10031681m|PACid:20802469 | 52117 | 665 | 27 | 23 | 12 | 8 | 5 | 1.515 | 0.93 | 1.199 | AT5G07440.1 | Glutamate dehydrogenase 2 |
| 769 | Ciclev10025269m|PACid:20800560 | 74961 | 225 | 12.6 | 15 | 3 | 5 | 3 | 1.517 | 1.307 | 1.269 | AT5G10240.1 | Asparagine synthetase 3 |
| 2485 | Ciclev10031794m|PACid:20804466 | 49939 | 109 | 10.6 | 6 | 6 | 4 | 4 | 1.521 | 0.833 | 1.025 | AT5G17710.2 | Co-chaperone GrpE family protein |
| 1499 | Ciclev10011534m|PACid:20797029 | 64202 | 299 | 17.7 | 16 | 16 | 7 | 7 | 1.532 | 1.287 | 0.97 | AT5G38530.1 | Tryptophan synthase beta type 2 |
| 1253 | Ciclev10004993m|PACid:20790333 | 52426 | 514 | 24.7 | 19 | 7 | 8 | 4 | 1.533 | 1.414 | 1.284 | AT1G03220.1 | Eukaryotic aspartyl protease family protein |
| 2048 | Ciclev10001338m|PACid:20787450 | 56222 | 77 | 6.9 | 3 | 3 | 2 | 2 | 1.538 | 1.008 | 1.082 | AT1G15200.1 | Protein-protein interaction regulator family protein |
| 2707 | Ciclev10005436m|PACid:20790220 | 42615 | 109 | 9.3 | 2 | 2 | 2 | 2 | 1.557 | 1.117 | 0.881 | AT1G12350.1 | 4-phospho-panto-thenoylcysteine synthetase |
| 499 | Ciclev10011525m|PACid:20798341 | 64142 | 170 | 8.1 | 10 | 10 | 4 | 4 | 1.566 | 0.893 | 1.067 | AT1G11910.1 | Aspartic proteinase A1 |
| 1086 | Ciclev10030299m|PACid:20812299 | 53479 | 87 | 9.6 | 4 | 2 | 3 | 1 | 1.566 | 1.227 | 0.926 | AT4G34740.1 | GLN phosphoribosyl pyrophosphate amidotransferase 2 |
| 2540 | Ciclev10007621m|PACid:20794872 | 88832 | 447 | 18.4 | 16 | 16 | 8 | 8 | 1.566 | 1.706 | 1.303 | AT5G19740.1 | Peptidase M28 family protein |
| 1850 | Ciclev10004182m|PACid:20790687 | 148436 | 149 | 7.1 | 8 | 6 | 6 | 5 | 1.59 | 1.664 | 1.332 | AT1G79570.1 | Protein kinase superfamily protein with octicosapeptide/Phox/Bem1p domain |
| 2059 | Ciclev10007313m|PACid:20795677 | 134981 | 157 | 8.5 | 10 | 10 | 8 | 8 | 1.594 | 1.061 | 1.133 | AT4G33150.1 | Lysine-ketoglutarate reductase/saccharopine dehydrogenase bifunctional enzyme |
| 2426 | Ciclev10007686m|PACid:20793409 | 83158 | 97 | 9.1 | 7 | 7 | 6 | 6 | 1.597 | 1.591 | 1.679 | AT1G56130.1 | Leucine-rich repeat transmembrane protein kinase |
| 92 | Ciclev10005038m|PACid:20790402 | 51272 | 505 | 37.6 | 25 | 20 | 12 | 9 | 1.603 | 1.512 | 1.06 | AT1G03220.1 | Eukaryotic aspartyl protease family protein |
| 2625 | Ciclev10030306m|PACid:20814141 | 60781 | 407 | 10.3 | 17 | 16 | 4 | 3 | 1.612 | 1.116 | 1.331 | AT5G64430.1 | Octicosapeptide/Phox/Bem1p family protein |
| 3303 | Ciclev10014821m|PACid:20816488 | 67047 | 63 | 7.7 | 3 | 3 | 3 | 3 | 1.627 | 0.769 | 1.206 | AT3G25660.1 | Amidase family protein |
| 318 | Ciclev10003175m|PACid:20787564 | 41477 | 49 | 4.6 | 2 | 2 | 1 | 1 | 1.628 | 1.322 | 1.208 | AT1G09130.1 | ATP-dependent caseinolytic (Clp) protease/crotonase family protein |
| 818 | Ciclev10025375m|PACid:20801560 | 66182 | 196 | 9.1 | 8 | 8 | 5 | 5 | 1.679 | 1.119 | 1.272 | AT5G65760.1 | Serine carboxypeptidase S28 family protein |
| 3192 | Ciclev10021900m|PACid:20810341 | 28689 | 144 | 29.8 | 7 | 4 | 5 | 2 | 1.683 | 1.05 | 1.207 | AT3G55620.1 | Translation initiation factor IF6 |
| 3127 | Ciclev10001559m|PACid:20786148 | 47035 | 400 | 9.8 | 10 | 10 | 2 | 2 | 1.781 | 1.17 | 1.329 | AT4G39090.1 | Papain family |
| 942 | Ciclev10015024m|PACid:20816530 | 63370 | 402 | 26.2 | 16 | 16 | 8 | 8 | 1.807 | 1.191 | 1.364 | AT3G63470.1 | Serine carboxypeptidase-like 40 |
| 1056 | Ciclev10019831m|PACid:20810707 | 67202 | 825 | 33.1 | 31 | 19 | 11 | 8 | 1.823 | 1.824 | 1.905 | AT3G17760.1 | glutamate decarboxylase 5 |
| 3215 | Ciclev10031853m|PACid:20804949 | 46394 | 88 | 9.4 | 5 | 5 | 4 | 4 | 1.94 | 1.223 | 0.917 | AT3G52180.1 | Dual specificity protein phosphatase (DsPTP1) family protein |
| 1217 | Ciclev10025194m|PACid:20800425 | 78264 | 61 | 11.8 | 6 | 6 | 5 | 5 | 1.961 | 1.144 | 1.344 | AT4G37670.2 | *N*-Acetylglutamate synthase 2 |
| 1240 | Ciclev10029224m|PACid:20812434 | 28670 | 71 | 8.8 | 5 | 5 | 2 | 2 | 2.001 | 1.069 | 1.151 | AT4G21860.1 | Methionine-S-oxide reductase B 2 |
| 2662 | Ciclev10002395m|PACid:20788045 | 29565 | 274 | 26.8 | 18 | 18 | 5 | 5 | 2.232 | 0.954 | 1.26 | AT1G17860.1 | Kunitz family trypsin and protease inhibitor protein |
| 2026 | Ciclev10028638m|PACid:20813396 | 50658 | 225 | 18.1 | 8 | 8 | 5 | 5 | 3.077 | 1.485 | 1.431 | AT1G49970.1 | CLP protease proteolytic subunit 1 |
| 2121 | Ciclev10027863m|PACid:20812235 | 95474 | 54 | 4.2 | 3 | 3 | 3 | 3 | 3.82 | 1.893 | 2.223 | AT5G51750.1 | Subtilase 1.3 |
| **Carbohydrate and energy metabolism** | |  |  |  |  |  |  |  |  |  |  |  |  |
| 2466 | Ciclev10019935m|PACid:20809278 | 65190 | 1673 | 23.8 | 58 | 11 | 9 | 1 | 0.431 | 0.573 | 0.635 | AT3G29360.1 | **UDP-glucose 6-dehydrogenase family protein** |
| 2731 | Ciclev10008809m|PACid:20795968 | 47082 | 484 | 24.1 | 25 | 25 | 7 | 7 | 0.473 | 0.847 | 0.693 | AT5G20400.1 | 2-oxoglutarate (2OG) and Fe(II)-dependent oxygenase superfamily protein |
| 1380 | Ciclev10032538m|PACid:20804612 | 32416 | 982 | 41.8 | 55 | 22 | 8 | 4 | 0.474 | 0.67 | 0.643 | AT3G55440.1 | Triosephosphate isomerase |
| 1700 | Ciclev10011622m|PACid:20796722 | 64909 | 3081 | 63.3 | 129 | 77 | 20 | 11 | 0.506 | 0.717 | 0.763 | AT5G15490.1 | UDP-glucose 6-dehydrogenase family protein |
| 2158 | Ciclev10001927m|PACid:20788381 | 43270 | 152 | 15.2 | 9 | 9 | 4 | 4 | 0.591 | 1.053 | 0.914 | AT1G77330.1 | 2-oxoglutarate (2OG) and Fe(II)-dependent oxygenase superfamily protein |
| 2666 | Ciclev10001399m|PACid:20785949 | 51785 | 88 | 8.8 | 4 | 4 | 3 | 3 | 0.644 | 0.862 | 0.679 | AT1G22440.1 | Zinc-binding alcohol dehydrogenase family protein |
| 257 | Ciclev10020160m|PACid:20808865 | 58906 | 260 | 26.2 | 26 | 21 | 12 | 9 | 0.653 | 0.885 | 0.819 | AT1G78860.1 | D-mannose binding lectin protein with Apple-like carbohydrate-binding domain |
| 501 | Ciclev10019180m|PACid:20810085 | 90542 | 1922 | 44.2 | 92 | 29 | 27 | 12 | 0.663 | 0.857 | 0.854 | AT1G78570.1 | Rhamnose biosynthesis 1 |
| 2363 | Ciclev10020328m|PACid:20806955 | 49331 | 232 | 10 | 10 | 10 | 3 | 3 | 0.759 | 1.671 | 0.866 | AT3G60900.1 | FASCICLIN-like arabinogalactan-protein 10 |
| 2438 | Ciclev10015925m|PACid:20818782 | 42275 | 162 | 21.8 | 8 | 8 | 6 | 6 | 1.134 | 0.474 | 1.74 | AT4G21580.1 | Oxidoreductase, zinc-binding dehydrogenase family protein |
| 212 | Ciclev10027083m|PACid:20799426 | 41987 | 59 | 10.4 | 4 | 4 | 3 | 3 | 1.504 | 1.068 | 1.381 | AT1G52790.1 | 2-oxoglutarate (2OG) and Fe(II)-dependent oxygenase superfamily protein |
| 2622 | Ciclev10005226m|PACid:20790515 | 50327 | 65 | 6.8 | 2 | 2 | 2 | 2 | 1.521 | 0.855 | 1.107 | AT1G06620.1 | 2-oxoglutarate (2OG) and Fe(II)-dependent oxygenase superfamily protein |
| 1701 | Ciclev10014829m|PACid:20815058 | 69902 | 329 | 16.8 | 17 | 10 | 9 | 6 | 1.522 | 1.073 | 1.001 | AT1G30760.1 | FAD-binding Berberine family protein |
| 2265 | Ciclev10011312m|PACid:20796268 | 80639 | 57 | 5.7 | 4 | 4 | 3 | 3 | 1.543 | 0.919 | 1.221 | AT1G31800.1 | Cytochrome P450, family 97, subfamily A, polypeptide 3 |
| 1637 | Ciclev10003261m|PACid:20789450 | 46885 | 389 | 32 | 17 | 6 | 9 | 4 | 1.546 | 1.598 | 1.693 | AT1G60690.1 | NAD(P)-linked oxidoreductase superfamily protein |
| 2846 | Ciclev10028560m|PACid:20813852 | 52161 | 129 | 20.7 | 7 | 7 | 6 | 6 | 1.548 | 1.162 | 0.958 | AT2G19940.1 | Oxidoreductases, acting on the aldehyde or oxo group of donors, NAD or NADP as acceptor;copper ion binding |
| 2428 | Ciclev10031821m|PACid:20805442 | 46553 | 207 | 22.3 | 12 | 7 | 7 | 5 | 1.551 | 1.297 | 0.998 | AT5G21060.2 | **Glyceraldehyde-3-phosphate dehydrogenase-like family protein** |
| 929 | Ciclev10021072m|PACid:20805955 | 44756 | 205 | 33.1 | 12 | 10 | 8 | 7 | 1.555 | 0.921 | 1.03 | AT4G13010.1 | Oxidoreductase, zinc-binding dehydrogenase family protein |
| 86 | Ciclev10028714m|PACid:20814721 | 47175 | 616 | 35.7 | 32 | 11 | 11 | 6 | 1.568 | 1.273 | 1.326 | AT4G35260.1 | **Isocitrate dehydrogenase 1** |
| 1893 | Ciclev10014947m|PACid:20819187 | 71622 | 86 | 6.4 | 7 | 7 | 3 | 3 | 1.576 | 0.974 | 1.259 | AT3G26300.1 | Cytochrome P450, family 71, subfamily B, polypeptide 34 |
| 2036 | Ciclev10025088m|PACid:20800760 | 89103 | 633 | 23.8 | 25 | 9 | 11 | 5 | 1.584 | 0.969 | 1.714 | AT4G37870.1 | Phosphoenolpyruvate carboxykinase 1 |
| 2852 | Ciclev10001689m|PACid:20787254 | 48840 | 334 | 14.9 | 15 | 10 | 4 | 2 | 1.601 | 1.304 | 1.158 | AT1G60690.1 | NAD(P)-linked oxidoreductase superfamily protein |
| 2380 | Ciclev10006451m|PACid:20791072 | 68967 | 287 | 18.4 | 19 | 11 | 10 | 6 | 1.602 | 1.073 | 0.967 | AT1G30760.1 | FAD-binding Berberine family protein |
| 1905 | Ciclev10028210m|PACid:20812178 | 67272 | 231 | 12.1 | 11 | 11 | 5 | 5 | 1.603 | 1.136 | 1.373 | AT2G28100.1 | Alpha-L-fucosidase 1 |
| 485 | Ciclev10027816m|PACid:20813550 | 110116 | 225 | 12.4 | 10 | 10 | 8 | 8 | 1.613 | 1.251 | 1.179 | AT4G34260.1 | 1,2-alpha-L-fucosidases |
| 2256 | Ciclev10032556m|PACid:20804067 | 34538 | 243 | 16.3 | 8 | 8 | 3 | 3 | 1.614 | 0.679 | 0.99 | AT2G38740.1 | Haloacid dehalogenase-like hydrolase (HAD) superfamily protein |
| 1516 | Ciclev10000811m|PACid:20785875 | 70693 | 179 | 12.7 | 7 | 4 | 5 | 3 | 1.624 | 1.258 | 1.136 | AT5G44400.1 | FAD-binding Berberine family protein |
| 2229 | Ciclev10021118m|PACid:20810224 | 44525 | 116 | 18 | 6 | 4 | 5 | 4 | 1.639 | 1.458 | 0.449 | AT4G13010.1 | Oxidoreductase, zinc-binding dehydrogenase family protein |
| 1308 | Ciclev10024910m|PACid:20801226 | 102498 | 112 | 4.3 | 4 | 4 | 3 | 3 | 1.643 | 1.692 | 1.434 | AT4G37270.1 | Heavy metal ATPase 1 |
| 262 | Ciclev10030338m|PACid:20813800 | 50263 | 49 | 4.9 | 2 | 2 | 2 | 2 | 1.672 | 1.119 | 1.266 | AT3G45740.1 | Hydrolase family protein / HAD-superfamily protein |
| 1534 | Ciclev10021069m|PACid:20811994 | 45529 | 266 | 12.3 | 14 | 14 | 4 | 4 | 1.676 | 1.299 | 1.275 | AT3G46490.1 | 2-oxoglutarate (2OG) and Fe(II)-dependent oxygenase superfamily protein |
| 2303 | Ciclev10015577m|PACid:20818784 | 51456 | 251 | 20.5 | 8 | 8 | 5 | 5 | 1.679 | 1.132 | 1.327 | AT1G03400.1 | 2-oxoglutarate (2OG) and Fe(II)-dependent oxygenase superfamily protein |
| 722 | Ciclev10011614m|PACid:20798380 | 69736 | 150 | 15.6 | 9 | 5 | 6 | 4 | 1.685 | 1.79 | 1.576 | AT5G40010.1 | AAA-ATPase 1 |
| 1520 | Ciclev10019998m|PACid:20807757 | 61045 | 112 | 14.4 | 6 | 6 | 5 | 5 | 1.687 | 1.26 | 1.281 | AT2G22590.1 | UDP-Glycosyltransferase superfamily protein |
| 1478 | Ciclev10029226m|PACid:20813808 | 31108 | 198 | 25.7 | 11 | 11 | 6 | 6 | 1.705 | 1.77 | 1.482 | AT1G16700.1 | Alpha-helical ferredoxin |
| 3109 | Ciclev10020878m|PACid:20811851 | 44124 | 280 | 21.6 | 13 | 13 | 4 | 4 | 1.722 | 1.347 | 1.177 | AT4G10750.1 | **Phosphoenolpyruvate carboxylase family protein** |
| 883 | Ciclev10019346m|PACid:20806605 | 80944 | 712 | 31.1 | 29 | 29 | 15 | 15 | 1.729 | 0.856 | 1.182 | AT1G32900.1 | UDP-Glycosyltransferase superfamily protein |
| 878 | Ciclev10005441m|PACid:20791236 | 40749 | 252 | 20 | 13 | 13 | 5 | 5 | 1.763 | 1.737 | 1.356 | AT5G62180.1 | Carboxyesterase 20 |
| 2760 | Ciclev10014929m|PACid:20814995 | 70690 | 226 | 16.6 | 12 | 12 | 8 | 8 | 1.773 | 1.155 | 1.135 | AT2G32290.1 | Beta-amylase 6 |
| 210 | Ciclev10027182m|PACid:20799633 | 42917 | 136 | 16.1 | 5 | 5 | 4 | 4 | 1.817 | 1.263 | 1.703 | AT1G52800.1 | 2-oxoglutarate (2OG) and Fe(II)-dependent oxygenase superfamily protein |
| 1764 | Ciclev10025490m|PACid:20799546 | 59430 | 113 | 4.5 | 4 | 4 | 2 | 2 | 1.818 | 1.332 | 1.642 | AT2G18570.1 | UDP-Glycosyltransferase superfamily protein |
| 2735 | Ciclev10004268m|PACid:20791548 | 116776 | 74 | 4.7 | 5 | 5 | 4 | 4 | 1.84 | 1.502 | 1.302 | AT2G32810.1 | **Beta galactosidase 9** |
| 856 | Ciclev10028769m|PACid:20813836 | 44906 | 1275 | 49.9 | 52 | 27 | 15 | 9 | 1.901 | 1.309 | 1.14 | AT1G60710.1 | NAD(P)-linked oxidoreductase superfamily protein |
| 1617 | Ciclev10028195m|PACid:20812602 | 70476 | 245 | 14 | 10 | 10 | 6 | 6 | 1.927 | 1.265 | 1.35 | AT4G39210.1 | Glucose-1-phosphate adenylyltransferase family protein |
| 2055 | Ciclev10018742m|PACid:20807145 | 122492 | 113 | 5.9 | 8 | 8 | 5 | 5 | 1.937 | 1.289 | 1.124 | AT5G49900.1 | Beta-glucosidase, GBA2 type family protein |
| 352 | Ciclev10005068m|PACid:20792308 | 56010 | 95 | 11.6 | 5 | 5 | 4 | 4 | 2.006 | 1.316 | 0.911 | AT1G04420.1 | NAD(P)-linked oxidoreductase superfamily protein |
| 1186 | Ciclev10000813m|PACid:20788807 | 69075 | 645 | 23.6 | 25 | 7 | 10 | 3 | 2.089 | 1.272 | 1.165 | AT4G20820.1 | FAD-binding Berberine family protein |
| 3305 | Ciclev10027805m|PACid:20814502 | 108357 | 311 | 10.7 | 16 | 11 | 7 | 5 | 2.177 | 1.283 | 1.675 | AT2G28470.1 | Beta-galactosidase 8 |
| 2179 | Ciclev10008095m|PACid:20793747 | 66967 | 52 | 4.9 | 2 | 2 | 2 | 2 | 2.306 | 1.069 | 1.616 | AT2G45550.1 | Cytochrome P450, family 76, subfamily C, polypeptide 4 |
| 14 | Ciclev10011544m|PACid:20798569 | 65109 | 372 | 19.8 | 13 | 13 | 7 | 7 | 2.313 | 1.138 | 1.267 | AT3G21760.1 | UDP-Glycosyltransferase superfamily protein |
| 1141 | Ciclev10012482m|PACid:20796497 | 34420 | 58 | 10.9 | 5 | 5 | 3 | 3 | 2.34 | 1.432 | 1.697 | AT3G02100.1 | UDP-Glycosyltransferase superfamily protein |
| 2496 | Ciclev10026096m|PACid:20800141 | 41913 | 108 | 13.4 | 3 | 3 | 3 | 3 | 2.605 | 0.794 | 1.416 | AT1G17710.1 | Pyridoxal phosphate phosphatase-related protein |
| 2257 | Ciclev10000256m|PACid:20786659 | 110995 | 160 | 9.7 | 8 | 8 | 7 | 7 | 2.733 | 1.036 | 1.345 | AT5G03650.1 | **Starch branching enzyme 2.2** |
| 3016 | Ciclev10009471m|PACid:20795826 | 29515 | 64 | 14.2 | 4 | 3 | 3 | 2 | 3.167 | 1.227 | 1.274 | AT1G59960.1 | NAD(P)-linked oxidoreductase superfamily protein |
| 241 | Ciclev10003556m|PACid:20788733 | 68060 | 829 | 21.1 | 30 | 14 | 9 | 4 | 3.222 | 1.598 | 1.576 | AT4G20820.1 | FAD-binding Berberine family protein |
| 1831 | Ciclev10032086m|PACid:20802454 | 43264 | 261 | 22.5 | 12 | 12 | 6 | 6 | 3.906 | 1.041 | 2.575 | AT5G06860.1 | Polygalacturonase inhibiting protein 1 |
| 921 | Ciclev10015897m|PACid:20815445 | 39667 | 651 | 35.9 | 32 | 32 | 10 | 10 | 4.039 | 1.184 | 2.625 | AT3G07720.1 | Galactose oxidase/kelch repeat superfamily protein |
| **Cellular transport** | |  |  |  |  |  |  |  |  |  |  |  |  |
| 984 | Ciclev10009426m|PACid:20793198 | 30850 | 668 | 40.3 | 31 | 31 | 8 | 8 | 0.549 | 0.783 | 0.756 | AT5G55190.1 | RAN GTPase 3 |
| 3284 | Ciclev10009163m|PACid:20795481 | 36513 | 1073 | 41.2 | 59 | 59 | 8 | 8 | 0.625 | 0.72 | 0.882 | AT5G57490.1 | Voltage dependent anion channel 4 |
| 1372 | Ciclev10016911m|PACid:20816266 | 23424 | 389 | 36.2 | 9 | 9 | 5 | 5 | 0.629 | 0.805 | 0.799 | AT1G60970.1 | SNARE-like superfamily protein |
| 508 | Ciclev10020020m|PACid:20810134 | 56648 | 71 | 3.8 | 2 | 2 | 1 | 1 | 0.586 | 0.702 | 0.647 | AT3G25150.2 | Nuclear transport factor 2 (NTF2) family protein with RNA binding (RRM-RBD-RNP motifs) domain |
| 2497 | Ciclev10020038m|PACid:20807970 | 56705 | 460 | 17.8 | 17 | 17 | 7 | 7 | 0.637 | 0.776 | 0.843 | AT5G60980.1 | Nuclear transport factor 2 (NTF2) family protein with RNA binding (RRM-RBD-RNP motifs) domain |
| 1135 | Ciclev10021502m|PACid:20805872 | 35799 | 95 | 8.4 | 3 | 3 | 2 | 2 | 0.653 | 0.679 | 0.873 | AT2G45960.1 | Plasma membrane intrinsic protein 1B |
| 3154 | Ciclev10011983m|PACid:20797349 | 49844 | 123 | 9.1 | 5 | 5 | 3 | 3 | 0.659 | 1.27 | 1.316 | AT4G17890.1 | ARF-GAP domain 8 |
| 1681 | Ciclev10000891m|PACid:20787766 | 65817 | 42 | 5.8 | 3 | 2 | 3 | 2 | 0.661 | 0.632 | 0.751 | AT2G01070.1 | Lung seven transmembrane receptor family protein |
| 2144 | Ciclev10005343m|PACid:20791520 | 46797 | 109 | 10.8 | 4 | 4 | 3 | 3 | 1.559 | 1.077 | 1.609 | AT5G51570.1 | SPFH/Band 7/PHB domain-containing membrane-associated protein family |
| 3213 | Ciclev10032182m|PACid:20805393 | 44728 | 80 | 14.8 | 4 | 4 | 4 | 4 | 1.576 | 1.24 | 1.389 | AT2G38750.1 | Annexin 4 |
| 795 | Ciclev10026128m|PACid:20801731 | 38850 | 114 | 25.1 | 5 | 4 | 5 | 4 | 1.599 | 1.244 | 1.778 | AT2G22500.1 | Uncoupling protein 5 |
| 1183 | Ciclev10022803m|PACid:20811598 | 17504 | 71 | 17.8 | 2 | 2 | 2 | 2 | 1.619 | 1.096 | 1.019 | AT1G80500.1 | SNARE-like superfamily protein |
| 1638 | Ciclev10000400m|PACid:20786370 | 87465 | 122 | 7.5 | 4 | 4 | 3 | 3 | 1.751 | 1.531 | 1.36 | AT4G35300.1 | Tonoplast monosaccharide transporter2 |
| 270 | Ciclev10007352m|PACid:20795667 | 128670 | 43 | 2.8 | 2 | 2 | 2 |  | 1.825 | 1.239 | 1.64 | AT5G24810.1 | ABC1 family protein |
| 778 | Ciclev10024720m|PACid:20802209 | 173465 | 112 | 3.4 | 4 | 4 | 3 | 3 | 1.839 | 1.176 | 1.448 | AT4G39850.1 | Peroxisomal ABC transporter 1 |
| 27 | Ciclev10026427m|PACid:20799682 | 29712 | 227 | 19.1 | 12 | 3 | 4 | 2 | 1.849 | 1.462 | 1.242 | AT5G65270.1 | RAB GTPase homolog A4A |
| 1547 | Ciclev10021867m|PACid:20807674 | 26896 | 138 | 6.4 | 3 | 3 | 1 | 1 | 2.165 | 1.934 | 1.722 | AT5G47450.1 | **Tonoplast intrinsic protein 2;3** |
| 1177 | Ciclev10000941m|PACid:20787191 | 57668 | 105 | 3.6 | 2 | 2 | 1 | 1 | 2.316 | 1.342 | 1.227 | AT1G09960.1 | Sucrose transporter 4 |
| 2842 | Ciclev10000425m|PACid:20787673 | 89447 | 677 | 28.4 | 36 | 36 | 17 | 17 | 2.349 | 1.091 | 1.332 | AT1G21680.1 | DPP6 N-terminal domain-like protein |
| 400 | Ciclev10009186m|PACid:20794673 | 35599 | 286 | 17.4 | 9 | 9 | 4 | 4 | 5.909 | 1.007 | 6.024 | AT1G67940.1 | **ABC transporter I family member 17** |
| **Cell wall and cytoskeleton metabolism** | |  |  |  |  |  |  |  |  |  |  |  |  |
| 83 | Ciclev10003323m|PACid:20786648 | 41415 | 49 | 3.4 | 3 | 3 | 1 | 1 | 0.29 | 0.455 | 0.572 | AT5G54160.1 | O-methyltransferase 1 |
| 1129 | Ciclev10013542m|PACid:20799197 | 46214 | 222 | 30.3 | 10 | 10 | 7 | 7 | 0.407 | 0.657 | 0.707 | AT5G54160.1 | O-methyltransferase 1 |
| 570 | Ciclev10011723m|PACid:20796428 | 55698 | 4623 | 64.3 | 212 | 14 | 23 | 4 | 0.558 | 0.948 | 0.866 | AT5G62690.1 | Tubulin beta chain 2 |
| 486 | Ciclev10025588m|PACid:20801298 | 56351 | 4774 | 56.8 | 143 | 4 | 18 | 1 | 0.586 | 0.927 | 0.871 | AT1G50010.1 | Tubulin alpha-2 chain |
| 2300 | Ciclev10015469m|PACid:20816426 | 52222 | 280 | 25.1 | 11 | 11 | 6 | 6 | 0.622 | 1.275 | 0.856 | AT4G12730.1 | FASCICLIN-like arabinogalactan 2 |
| 1590 | Ciclev10011709m|PACid:20797581 | 56340 | 3840 | 49.3 | 118 | 2 | 16 | 2 | 0.629 | 0.844 | 0.824 | AT1G50010.1 | Tubulin alpha-2 chain |
| 2644 | Ciclev10011719m|PACid:20798687 | 55193 | 4727 | 64 | 202 | 15 | 23 | 5 | 0.633 | 0.82 | 0.784 | AT5G12250.1 | Beta-6 tubulin |
| 2600 | Ciclev10015224m|PACid:20818962 | 55822 | 4398 | 52.6 | 132 | 14 | 17 | 2 | 0.646 | 1.02 | 0.893 | AT5G19770.1 | Tubulin alpha-3 |
| 1435 | Ciclev10032221m|PACid:20804536 | 38657 | 511 | 33.4 | 23 | 23 | 7 | 7 | 0.663 | 0.799 | 0.818 | AT4G25630.1 | Fibrillarin 2 |
| 2772 | Ciclev10026361m|PACid:20801792 | 31989 | 178 | 18 | 11 | 3 | 4 | 1 | 1.211 | 1.734 | 0.711 | AT5G54160.1 | O-methyltransferase 1 |
| 1698 | Ciclev10012166m|PACid:20798963 | 43761 | 146 | 26 | 8 | 8 | 5 | 5 | 1.513 | 1.354 | 1.396 | AT2G37660.1 | NAD(P)-binding Rossmann-fold superfamily protein |
| 51 | Ciclev10028731m|PACid:20813451 | 48435 | 286 | 27.7 | 12 | 12 | 7 | 7 | 1.552 | 1.274 | 1.524 | AT4G35160.1 | O-methyltransferase family protein |
| 3211 | Ciclev10011375m|PACid:20798385 | 69035 | 148 | 6.3 | 8 | 5 | 4 | 3 | 1.558 | 0.863 | 1.382 | AT3G09220.1 | Laccase 7 |
| 1784 | Ciclev10019941m|PACid:20806533 | 60502 | 241 | 11 | 7 | 7 | 3 | 3 | 1.564 | 1.191 | 1.308 | AT4G23500.1 | Pectin lyase-like superfamily protein |
| 1935 | Ciclev10005275m|PACid:20792294 | 47334 | 1115 | 58.7 | 50 | 15 | 15 | 6 | 1.576 | 1.046 | 1.519 | AT4G35160.1 | O-methyltransferase family protein |
| 2605 | Ciclev10002233m|PACid:20786426 | 31451 | 75 | 12.9 | 7 | 7 | 3 | 3 | 1.662 | 1.021 | 1.243 | AT1G20190.1 | Expansin 11 |
| 2498 | Ciclev10022142m|PACid:20811428 | 28369 | 1101 | 31.2 | 33 | 15 | 4 | 2 | 1.743 | 1.217 | 1.642 | AT5G39120.1 | RmlC-like cupins superfamily protein |
| 399 | Ciclev10020108m|PACid:20809617 | 55598 | 60 | 8 | 2 | 2 | 2 | 2 | 1.906 | 1.111 | 1.046 | AT1G02810.1 | Plant invertase/pectin methylesterase inhibitor superfamily |
| 686 | Ciclev10021884m|PACid:20807416 | 29804 | 192 | 22.3 | 6 | 6 | 3 | 3 | 1.95 | 1.682 | 1.356 | AT4G17030.1 | Expansin-like B1 |
| 291 | Ciclev10012221m|PACid:20797915 | 41199 | 193 | 17.8 | 9 | 4 | 4 | 3 | 1.97 | 1.306 | 1.512 | AT2G29290.2 | NAD(P)-binding Rossmann-fold superfamily protein |
| 710 | Ciclev10015708m|PACid:20818094 | 49104 | 311 | 31.5 | 19 | 16 | 9 | 8 | 2.369 | 1.303 | 1.725 | AT4G35160.1 | O-methyltransferase family protein |
| 193 | Ciclev10032524m|PACid:20804072 | 29630 | 62 | 3.5 | 4 | 4 | 1 | 1 | 2.938 | 1.222 | 1.078 | AT2G40610.1 | Expansin A8 |
| 1729 | Ciclev10022050m|PACid:20809017 | 30713 | 85 | 16.9 | 6 | 4 | 4 | 3 | 3.468 | 1.225 | 1.382 | AT3G54420.1 | Homolog of carrot EP3-3 chitinase |
| 53 | Ciclev10006669m|PACid:20789722 | 28918 | 262 | 15.1 | 23 | 4 | 4 | 1 | 5.04 | 2.56 | 3.454 | AT1G50010.1 | Tubulin alpha-2 chain |
| 1997 | Ciclev10022152m|PACid:20806911 | 27941 | 384 | 11.8 | 13 | 4 | 2 | 1 | 5.759 | 1.411 | 1.735 | AT5G39120.1 | RmlC-like cupins superfamily protein |
| 1907 | Ciclev10023297m|PACid:20807023 | 29607 | 115 | 10.4 | 5 | 3 | 2 | 1 | 6.825 | 1.297 | 1.565 | AT3G54420.1 | Homolog of carrot EP3-3 chitinase |
| 1925 | Ciclev10028959m|PACid:20812898 | 35485 | 268 | 11.3 | 16 | 12 | 3 | 2 | 9.403 | 1.603 | 2.296 | AT3G12500.1 | Basic chitinase |
| **Stress response** | |  |  |  |  |  |  |  |  |  |  |  |  |
| 682 | Ciclev10032680m|PACid:20805511 | 32181 | 450 | 19.1 | 23 | 18 | 5 | 3 | 0.461 | 0.758 | 0.918 | AT1G10370.1 | **Glutathione S-transferase family protein** |
| 2879 | Ciclev10033622m|PACid:20803991 | 87637 | 5931 | 40.9 | 215 | 13 | 21 | 2 | 0.537 | 0.873 | 0.748 | AT5G02500.1 | Heat shock cognate protein 70-1 |
| 416 | Ciclev10014579m|PACid:20818888 | 87220 | 44 | 2.8 | 3 | 3 | 2 | 2 | 0.565 | 0.777 | 0.416 | AT3G01420.1 | Peroxidase superfamily protein |
| 2222 | Ciclev10012361m|PACid:20797482 | 38118 | 91 | 11 | 3 | 3 | 2 | 2 | 0.579 | 0.87 | 0.897 | AT3G12130.1 | KH domain-containing protein / zinc finger (CCCH type) family protein |
| 2160 | Ciclev10033058m|PACid:20804847 | 18132 | 128 | 29.5 | 6 | 6 | 3 | 3 | 0.594 | 0.581 | 0.794 | AT5G42850.1 | Thioredoxin superfamily protein |
| 2717 | Ciclev10022431m|PACid:20807475 | 26604 | 369 | 30.3 | 18 | 18 | 4 | 4 | 0.653 | 0.818 | 0.775 | AT4G02450.1 | HSP20-like chaperones superfamily protein |
| 2870 | Ciclev10016752m|PACid:20815007 | 25968 | 1933 | 55.7 | 102 | 33 | 9 | 4 | 0.653 | 0.999 | 0.852 | AT4G27270.1 | Quinone reductase family protein |
| 3334 | Ciclev10022218m|PACid:20809718 | 28689 | 90 | 5.6 | 2 | 2 | 1 | 1 | 0.694 | 0.548 | 1.116 | AT3G62580.1 | Late embryogenesis abundant protein (LEA) family protein |
| 2322 | Ciclev10032743m|PACid:20802959 | 30625 | 466 | 25.1 | 21 | 7 | 5 | 2 | 1.513 | 1.107 | 1.402 | AT2G30860.1 | Glutathione S-transferase PHI 9 |
| 29 | Ciclev10015774m|PACid:20815197 | 47553 | 255 | 27 | 15 | 15 | 8 | 8 | 1.515 | 1.157 | 1.209 | AT4G19880.1 | Glutathione S-transferase family protein |
| 3047 | Ciclev10008605m|PACid:20794685 | 50284 | 77 | 8.5 | 4 | 2 | 3 | 2 | 1.515 | 1.23 | 1.378 | AT5G19460.1 | Nudix hydrolase homolog 20 |
| 529 | Ciclev10028103m|PACid:20812766 | 73349 | 135 | 19.3 | 8 | 8 | 7 | 7 | 1.524 | 1.391 | 1.323 | AT4G34030.1 | 3-methylcrotonyl-CoA carboxylase |
| 2263 | Ciclev10032471m|PACid:20804478 | 35949 | 422 | 36.6 | 13 | 13 | 6 | 6 | 1.531 | 0.681 | 1.238 | AT2G40300.1 | Ferritin 4 |
| 1123 | Ciclev10007294m|PACid:20793995 | 154265 | 426 | 14 | 23 | 13 | 12 | 8 | 1.534 | 1.211 | 1.226 | AT4G27190.1 | NB-ARC domain-containing disease resistance protein |
| 1868 | Ciclev10029149m|PACid:20813860 | 29754 | 423 | 23.4 | 18 | 18 | 5 | 5 | 1.548 | 1.073 | 0.952 | AT2G28790.1 | Pathogenesis-related thaumatin superfamily protein |
| 566 | Ciclev10020281m|PACid:20809559 | 53189 | 231 | 21.9 | 9 | 9 | 6 | 6 | 1.556 | 1.207 | 1.465 | AT5G54140.1 | IAA-leucine-resistant (ILR1)-like 3 |
| 647 | Ciclev10021197m|PACid:20809248 | 40664 | 83 | 10.9 | 4 | 4 | 3 | 3 | 1.575 | 1.05 | 1.54 | AT4G39460.1 | S-adenosylmethionine carrier 1 |
| 233 | Ciclev10006637m|PACid:20792180 | 161460 | 65 | 4 | 4 | 3 | 4 | 3 | 1.588 | 1.008 | 2.381 | AT1G02520.1 | P-glycoprotein 11 |
| 6 | Ciclev10010808m|PACid:20795663 | 38373 | 89 | 8.6 | 4 | 4 | 2 | 2 | 1.59 | 1.302 | 1.065 | AT4G29830.1 | Transducin/WD40 repeat-like superfamily protein |
| 1136 | Ciclev10032737m|PACid:20804434 | 30555 | 261 | 12.7 | 18 | 4 | 4 | 1 | 1.63 | 1.28 | 1.636 | AT2G30860.1 | Glutathione S-transferase PHI 9 |
| 2780 | Ciclev10018532m|PACid:20808164 | 159969 | 227 | 8.9 | 7 | 6 | 7 | 6 | 1.645 | 1.214 | 1.12 | AT1G02520.1 | P-glycoprotein 11 |
| 333 | Ciclev10014196m|PACid:20818257 | 117677 | 85 | 5.1 | 5 | 4 | 4 | 3 | 1.57 | 1.555 | 1.548 | AT5G11720.1 | Glycosyl hydrolases family 31 protein |
| 2060 | Ciclev10014123m|PACid:20816336 | 134119 | 454 | 13.5 | 20 | 11 | 11 | 7 | 1.653 | 1.218 | 1.08 | AT5G13980.1 | Glycosyl hydrolase family 38 protein |
| 916 | Ciclev10004509m|PACid:20789695 | 82493 | 166 | 15.5 | 10 | 7 | 8 | 6 | 1.724 | 0.913 | 1.048 | AT5G12950.1 | Glycosyl hydrolase of unknown function (DUF1680) |
| 1313 | Ciclev10014240m|PACid:20818942 | 111990 | 150 | 8.4 | 7 | 4 | 7 | 4 | 1.78 | 1.205 | 1.279 | AT5G12950.1 | Glycosyl hydrolase of unknown function (DUF1680) |
| 2593 | Ciclev10017635m|PACid:20816899 | 96285 | 292 | 14.4 | 18 | 15 | 8 | 7 | 1.862 | 1.205 | 1.368 | AT3G26720.1 | **Glycosyl hydrolase family 38 protein** |
| 528 | Ciclev10028372m|PACid:20812271 | 56201 | 109 | 15.4 | 6 | 6 | 5 | 5 | 2.027 | 0.941 | 1.477 | AT4G34480.1 | Glycosyl hydrolases family 17 protein |
| 1208 | Ciclev10010227m|PACid:20793090 | 44806 | 65 | 7.3 | 3 | 3 | 2 | 2 | 2.095 | 1.693 | 1.138 | AT4G19810.1 | Glycosyl hydrolase family protein with chitinase insertion domain |
| 1095 | Ciclev10000420m|PACid:20788618 | 91495 | 43 | 3.2 | 2 | 2 | 2 | 2 | 2.167 | 1.105 | 1.29 | AT5G63800.1 | Glycosyl hydrolase family 35 protein |
| 1844 | Ciclev10008669m|PACid:20793102 | 45833 | 68 | 9.6 | 4 | 4 | 3 | 3 | 2.407 | 1.428 | 1.463 | AT4G19810.1 | Glycosyl hydrolase family protein with chitinase insertion domain |
| 1373 | Ciclev10027796m|PACid:20813788 | 99979 | 79 | 1.9 | 5 | 5 | 1 | 1 | 2.418 | 2.052 | 1.816 | AT5G58480.1 | Glycosyl hydrolases family 17 protein |
| 96 | Ciclev10032510m|PACid:20802930 | 34384 | 414 | 16.3 | 16 | 11 | 4 | 3 | 1.655 | 0.99 | 1 | AT3G25780.1 | Allene oxide cyclase 3 |
| 2066 | Ciclev10024933m|PACid:20800377 | 104140 | 471 | 16.9 | 19 | 19 | 11 | 11 | 1.702 | 1.275 | 1.066 | AT1G79690.1 | Nudix hydrolase homolog 3 |
| 746 | Ciclev10006099m|PACid:20791868 | 23270 | 1038 | 42.9 | 31 | 31 | 6 | 6 | 1.708 | 1.232 | 1.766 | AT1G24020.1 | MLP-like protein 423 |
| 2174 | Ciclev10009304m|PACid:20794954 | 34233 | 147 | 22 | 8 | 4 | 4 | 3 | 1.71 | 1.107 | 1.157 | AT4G31870.1 | **Glutathione peroxidase 7** |
| 2135 | Ciclev10030519m|PACid:20803054 | 172637 | 111 | 5.5 | 5 | 5 | 5 | 5 | 1.751 | 1.412 | 0.999 | AT3G55320.1 | P-glycoprotein 20 |
| 801 | Ciclev10022164m|PACid:20810119 | 33056 | 288 | 22.4 | 20 | 15 | 6 | 4 | 1.779 | 1.366 | 1.242 | AT1G78380.1 | Glutathione S-transferase TAU 19 |
| 1740 | Ciclev10033001m|PACid:20802495 | 21533 | 82 | 18.4 | 2 | 2 | 1 | 1 | 1.874 | 1.521 | 1.461 | AT2G30870.1 | Glutathione S-transferase PHI 10 |
| 814 | Ciclev10032686m|PACid:20805199 | 33050 | 71 | 24.4 | 5 | 5 | 5 | 5 | 1.877 | 1.463 | 1.466 | AT2G29420.1 | Glutathione S-transferase tau 7 |
| 349 | Ciclev10012170m|PACid:20796979 | 42267 | 590 | 36.4 | 22 | 22 | 9 | 9 | 1.909 | 1.072 | 1.414 | AT2G37130.1 | Peroxidase superfamily protein |
| 3065 | Ciclev10015779m|PACid:20818417 | 28443 | 1021 | 36 | 34 | 7 | 5 | 3 | 1.938 | 0.882 | 1.111 | AT5G06720.1 | Peroxidase 2 |
| 2627 | Ciclev10015905m|PACid:20817929 | 42978 | 315 | 20.7 | 11 | 11 | 5 | 5 | 2.046 | 0.916 | 1.906 | AT2G01890.1 | Purple acid phosphatase 8 |
| 403 | Ciclev10016043m|PACid:20818538 | 36587 | 246 | 23.1 | 6 | 6 | 4 | 4 | 2.567 | 1.912 | 1.643 | AT5G14130.1 | Peroxidase superfamily protein |
| 759 | Ciclev10012621m|PACid:20797419 | 34826 | 202 | 31.9 | 11 | 7 | 6 | 4 | 2.696 | 1.244 | 1.981 | AT5G02790.1 | Glutathione S-transferase family protein |
| 2145 | Ciclev10021720m|PACid:20810657 | 35467 | 194 | 16.2 | 8 | 8 | 4 | 4 | 3.089 | 1.265 | 1.526 | AT2G41380.1 | S-adenosyl-L-methionine-dependent methyltransferases superfamily protein |
| 1712 | Ciclev10012473m|PACid:20797152 | 32977 | 226 | 11.6 | 5 | 5 | 2 | 2 | 3.218 | 1.243 | 3.133 | AT2G37330.1 | **Aluminum sensitive 3** |
| 779 | Ciclev10022104m|PACid:20810955 | 28016 | 88 | 5.8 | 2 | 2 | 1 | 1 | 4.741 | 1.946 | 1.422 | AT4G11650.1 | Osmotin 34 |
| 3261 | Ciclev10029712m|PACid:20812742 | 11418 | 62 | 13.4 | 3 | 3 | 1 | 1 | 5.656 | 0.423 | 1.316 | AT1G19610.1 | *Arabidopsis* defensin-like protein |
| **Lipid metabolism** | |  |  |  |  |  |  |  |  |  |  |  |  |
| 2681 | Ciclev10005918m|PACid:20791870 | 29050 | 307 | 35.1 | 24 | 24 | 6 | 6 | 0.649 | 0.828 | 0.77 | AT2G33470.1 | Glycolipid transfer protein 1 |
| 2858 | Ciclev10002299m|PACid:20789340 | 29764 | 177 | 19.7 | 5 | 5 | 3 | 3 | 0.654 | 0.928 | 0.686 | AT4G34150.1 | Calcium-dependent lipid-binding (CaLB domain) family protein |
| 1874 | Ciclev10026080m|PACid:20799388 | 43838 | 125 | 10.2 | 10 | 10 | 3 | 3 | 0.66 | 0.752 | 0.874 | AT1G52760.1 | Lysophospholipase 2 |
| 1827 | Ciclev10015302m|PACid:20819212 | 59623 | 800 | 36.3 | 41 | 41 | 13 | 13 | 0.771 | 0.63 | 1.016 | AT5G41040.1 | HXXXD-type acyl-transferase family protein |
| 605 | Ciclev10008993m|PACid:20794750 | 43989 | 189 | 13.2 | 7 | 7 | 3 | 3 | 1.172 | 1.557 | 0.818 | AT2G19590.1 | ACC oxidase 1 |
| 2781 | Ciclev10004113m|PACid:20791434 | 508940 | 44 | 1.1 | 4 | 4 | 4 | 4 | 1.591 | 1.666 | 1.062 | AT1G48090.1 | Calcium-dependent lipid-binding family protein |
| 1273 | Ciclev10009222m|PACid:20794980 | 35721 | 171 | 23.3 | 10 | 10 | 5 | 5 | 1.594 | 1.03 | 1.204 | AT4G29260.1 | HAD superfamily, subfamily IIIB acid phosphatase |
| 2492 | Ciclev10005338m|PACid:20791554 | 42981 | 136 | 7.6 | 5 | 5 | 2 | 2 | 1.678 | 1.015 | 1.32 | AT5G62180.1 | Carboxyesterase 20 |
| 1525 | Ciclev10026538m|PACid:20800794 | 23981 | 688 | 24.9 | 15 | 15 | 3 | 3 | 1.704 | 1.214 | 1.209 | AT2G22170.1 | Lipooxygenase, PLAT/LH2 family protein |
| 2595 | Ciclev10011796m|PACid:20797859 | 57480 | 79 | 6.6 | 5 | 5 | 3 | 3 | 1.836 | 1.169 | 1.394 | AT1G24430.1 | HXXXD-type acyl-transferase family protein |
| 2063 | Ciclev10026950m|PACid:20802000 | 64015 | 162 | 12.5 | 5 | 5 | 4 | 4 | 1.993 | 1.087 | 1.143 | AT3G26040.1 | HXXXD-type acyl-transferase family protein |
| 1113 | Ciclev10014212m|PACid:20816315 | 119111 | 3162 | 43.1 | 154 | 112 | 32 | 24 | 2.278 | 1.061 | 1.14 | AT3G45140.1 | **Lipoxygenase 2** |
| 1055 | Ciclev10014202m|PACid:20819066 | 120200 | 337 | 6.4 | 25 | 2 | 6 | 1 | 2.297 | 0.911 | 1.059 | AT3G45140.1 | Lipoxygenase 2 |
| 2235 | Ciclev10007095m|PACid:20790772 | 35730 | 216 | 15.1 | 5 | 5 | 3 | 3 | 2.394 | 0.994 | 1.549 | AT3G23400.1 | Plastid-lipid associated protein PAP / fibrillin family protein |
| 2501 | Ciclev10026223m|PACid:20801128 | 37398 | 87 | 11.7 | 5 | 5 | 3 | 3 | 2.665 | 0.825 | 0.991 | AT5G51260.1 | HAD superfamily, subfamily IIIB acid phosphatase |
| 1778 | Ciclev10014209m|PACid:20815705 | 123040 | 212 | 3.4 | 17 | 8 | 4 | 2 | 2.752 | 1.068 | 1.074 | AT3G45140.1 | Lipoxygenase 2 |
| **Nuclear acid metabolism** | |  |  |  |  |  |  |  |  |  |  |  |  |
| 1150 | Ciclev10002262m|PACid:20785551 | 34612 | 70 | 10.4 | 6 | 6 | 3 | 3 | 0.445 | 1.092 | 0.45 | AT5G64200.1 | Arginine/serine-rich splicing factor SC35 |
| 925 | Ciclev10027732m|PACid:20814761 | 144371 | 81 | 2 | 3 | 3 | 2 | 2 | 0.517 | 1.056 | 0.794 | AT3G19670.1 | **Pre-mRNA-processing protein 40B** |
| 2084 | Ciclev10017167m|PACid:20817475 | 17349 | 81 | 10.9 | 3 | 3 | 1 | 1 | 0.633 | 0.704 | 0.82 | AT1G29850.2 | dsDNA-binding family protein |
| 2183 | Ciclev10022481m|PACid:20811565 | 23850 | 722 | 33.7 | 26 | 12 | 6 | 4 | 0.658 | 1.006 | 0.92 | AT4G25740.1 | RNA binding Plectin/S10 domain-containing protein |
| 780 | Ciclev10016242m|PACid:20815326 | 34941 | 310 | 16.5 | 11 | 11 | 3 | 3 | 1.328 | 0.663 | 1.109 | AT2G39780.2 | Ribonuclease 2 |
| 8 | Ciclev10011787m|PACid:20798680 | 57659 | 91 | 8.4 | 3 | 3 | 3 | 3 | 1.509 | 1.358 | 1.246 | AT3G58140.1 | Phenylalanyl-tRNA synthetase class II family protein |
| 642 | Ciclev10023768m|PACid:20808527 | 42040 | 103 | 8.5 | 4 | 2 | 2 | 1 | 1.512 | 1.265 | 1.302 | AT1G32790.1 | CTC-interacting domain 11 |
| 1855 | Ciclev10027983m|PACid:20812527 | 82453 | 231 | 19.4 | 9 | 9 | 8 | 8 | 1.555 | 1.726 | 1.261 | AT2G37690.1 | Phosphoribosyl aminoimidazole carboxylase, putative / AIR carboxylase, putative |
| 2573 | Ciclev10009699m|PACid:20794339 | 20747 | 104 | 29.1 | 5 | 5 | 4 | 4 | 1.567 | 1.001 | 1.311 | AT5G56260.1 | Ribonuclease E inhibitor RraA/Dimethylmenaquinone methyltransferase |
| 634 | Ciclev10032674m|PACid:20804054 | 32147 | 190 | 15.9 | 12 | 12 | 4 | 4 | 1.675 | 1.005 | 1.476 | AT1G26820.1 | Ribonuclease 3 |
| 2916 | Ciclev10008107m|PACid:20794006 | 64736 | 57 | 9.5 | 3 | 3 | 3 | 3 | 1.91 | 1.257 | 1.308 | AT2G31170.1 | Cysteinyl-tRNA synthetase, class I family protein |
| **Biological regulation and signal transduction** | |  |  |  |  |  |  |  |  |  |  |  |  |
| 2081 | Ciclev10001298m|PACid:20786736 | 62868 | 1140 | 40 | 64 | 64 | 12 | 12 | 0.53 | 1.034 | 0.819 | AT1G56340.1 | Calreticulin-1 |
| 1109 | Ciclev10016127m|PACid:20818296 | 36799 | 310 | 21.9 | 8 | 8 | 3 | 3 | 0.546 | 0.903 | 0.939 | AT1G30630.1 | Coatomer epsilon subunit |
| 1402 | Ciclev10031861m|PACid:20802522 | 50103 | 238 | 15.5 | 17 | 17 | 5 | 5 | 0.578 | 0.898 | 0.833 | AT5G08580.1 | Calcium-binding EF hand family protein |
| 828 | Ciclev10022016m|PACid:20807234 | 33627 | 73 | 11.5 | 3 | 3 | 2 | 2 | 0.612 | 0.831 | 0.85 | AT1G01820.1 | Peroxin 11c |
| 732 | Ciclev10016204m|PACid:20815170 | 35796 | 88 | 4.3 | 3 | 3 | 1 | 1 | 0.621 | 0.817 | 0.954 | AT3G21865.1 | Peroxin 22 |
| 2841 | Ciclev10000859m|PACid:20785977 | 65758 | 178 | 11.7 | 7 | 7 | 5 | 5 | 1.521 | 0.963 | 1.179 | AT3G10660.1 | Calmodulin-domain protein kinase cdpk isoform 2 |
| 1761 | Ciclev10004218m|PACid:20789960 | 139227 | 131 | 7.6 | 5 | 4 | 5 | 4 | 1.574 | 0.943 | 1.118 | AT2G18790.1 | Phytochrome B |
| 2569 | Ciclev10011052m|PACid:20798944 | 113197 | 68 | 3.8 | 2 | 2 | 2 | 2 | 1.583 | 1.28 | 1.181 | AT5G06680.1 | Spindle pole body component 98 |
| 1479 | Ciclev10001032m|PACid:20786351 | 55293 | 136 | 7.6 | 3 | 3 | 3 | 3 | 1.656 | 0.998 | 1.013 | AT1G54730.2 | Major facilitator superfamily protein |
| 2465 | Ciclev10026050m|PACid:20799673 | 42813 | 147 | 9.4 | 3 | 3 | 2 | 2 | 1.698 | 1.285 | 1.304 | AT5G67400.1 | Root hair specific 19 |
| 299 | Ciclev10004220m|PACid:20791062 | 154767 | 40 | 2.3 | 2 | 2 | 2 | 2 | 1.712 | 1.28 | 1.132 | AT3G06400.2 | Chromatin-remodeling protein 11 |
| 1254 | Ciclev10019466m|PACid:20810070 | 75189 | 127 | 13 | 6 | 6 | 5 | 5 | 1.728 | 1.491 | 1.296 | AT1G28130.1 | Auxin-responsive GH3 family protein |
| 1776 | Ciclev10026610m|PACid:20801880 | 23423 | 69 | 13.1 | 3 | 3 | 2 | 2 | 1.728 | 1.455 | 1.386 | AT3G18430.1 | Calcium-binding EF-hand family protein |
| 1692 | Ciclev10032615m|PACid:20804275 | 29993 | 306 | 17.6 | 13 | 13 | 4 | 4 | 1.731 | 1.195 | 1.277 | AT3G07390.1 | Auxin-responsive family protein |
| 2639 | Ciclev10005981m|PACid:20790565 | 24592 | 61 | 5.8 | 2 | 2 | 1 | 1 | 1.753 | 1.249 | 1.357 | AT5G53160.2 | Regulatory components of ABA receptor 3 |
| 3149 | Ciclev10026660m|PACid:20801578 | 20657 | 154 | 21.5 | 5 | 4 | 3 | 2 | 2.032 | 0.961 | 1.432 | AT5G62200.1 | Embryo-specific protein 3 (ATS3) |
| 1445 | Ciclev10029937m|PACid:20813082 | 24647 | 153 | 10.7 | 7 | 7 | 2 | 2 | 2.61 | 3.851 | 1.77 | AT3G62020.1 | Germin-like protein 10 |
| 1849 | Ciclev10014250m|PACid:20818943 | 108910 | 44 | 3.5 | 2 | 2 | 2 | 2 | 1.587 | 1.65 | 1.435 | AT1G30570.1 | HERCULES receptor kinase 2 |
| 2073 | Ciclev10031585m|PACid:20803827 | 59885 | 320 | 30.3 | 20 | 18 | 11 | 10 | 1.614 | 1.361 | 1.173 | AT1G14000.1 | VH1-interacting kinase |
| **Other biological process** | |  |  |  |  |  |  |  |  |  |  |  |  |
| 1361 | Ciclev10021398m|PACid:20808210 | 38353 | 524 | 36.4 | 24 | 4 | 8 | 2 | 0.392 | 0.901 | 1.184 | AT4G02860.1 | Phenazine biosynthesis PhzC/PhzF protein |
| 2928 | Ciclev10019700m|PACid:20809599 | 70695 | 62 | 1.3 | 5 | 5 | 1 | 1 | 0.48 | 0.768 | 0.934 | AT1G06800.1 | Alpha/beta-Hydrolases superfamily protein |
| 1080 | Ciclev10012788m|PACid:20797116 | 31031 | 103 | 13.1 | 10 | 10 | 4 | 4 | 0.555 | 0.77 | 0.617 | AT1G57860.1 | Translation protein SH3-like family protein |
| 2572 | Ciclev10013125m|PACid:20798460 | 16434 | 249 | 43.8 | 12 | 12 | 4 | 4 | 0.584 | 0.733 | 0.75 | AT5G01650.2 | Tautomerase/MIF superfamily protein |
| 2273 | Ciclev10014215m|PACid:20816968 | 102321 | 183 | 8.2 | 6 | 6 | 5 | 5 | 0.595 | 0.982 | 1.094 | AT3G07660.1 | Kinase-related protein of unknown function (DUF1296) |
| 488 | Ciclev10026693m|PACid:20800442 | 20479 | 54 | 18.2 | 2 | 2 | 2 | 2 | 0.601 | 1.047 | 0.69 | AT5G66170.3 | Sulfurtransferase 18 |
| 2836 | Ciclev10022283m|PACid:20810800 | 32536 | 156 | 18.9 | 11 | 11 | 4 | 4 | 0.601 | 0.804 | 0.548 | AT3G49010.1 | Breast basic conserved 1 |
| 1900 | Ciclev10011817m|PACid:20797933 | 55821 | 205 | 13.3 | 7 | 7 | 4 | 4 | 0.63 | 0.594 | 0.825 | AT5G39570.1 | Unknown protein |
| 2216 | Ciclev10030821m|PACid:20804732 | 91550 | 2091 | 39.5 | 87 | 51 | 22 | 12 | 0.637 | 0.946 | 0.776 | AT2G37040.1 | PHE ammonia lyase 1 |
| 1408 | Ciclev10028604m|PACid:20813991 | 52634 | 525 | 27.6 | 25 | 16 | 8 | 6 | 0.64 | 0.81 | 0.834 | AT5G13930.1 | Chalcone and stilbene synthase family protein |
| 1596 | Ciclev10022849m|PACid:20811950 | 17016 | 91 | 18.8 | 4 | 4 | 2 | 2 | 0.648 | 0.862 | 0.95 | AT4G01897.1 | Unknown protein |
| 3216 | Ciclev10030093m|PACid:20813508 | 51250 | 1308 | 28.3 | 43 | 37 | 8 | 7 | 0.656 | 0.859 | 0.747 | AT5G13930.1 | Chalcone and stilbene synthase family protein |
| 2576 | Ciclev10019348m|PACid:20811777 | 81323 | 61 | 6.1 | 4 | 4 | 3 | 3 | 0.662 | 1.023 | 0.798 | AT3G13460.1 | Evolutionarily conserved C-terminal region 2 |
| 1063 | Ciclev10022444m|PACid:20810132 | 23449 | 136 | 29.6 | 8 | 8 | 4 | 4 | 1.505 | 1.063 | 0.987 | AT1G71310.1 | Cobalt ion binding |
| 2872 | Ciclev10011628m|PACid:20798109 | 60070 | 131 | 19.2 | 7 | 7 | 6 | 6 | 1.514 | 0.84 | 1.134 | AT3G52610.1 | Unknown protein |
| 2584 | Ciclev10028755m|PACid:20813771 | 42080 | 88 | 12.5 | 4 | 2 | 3 | 2 | 1.528 | 1.441 | 1.008 | AT5G22300.1 | Nitrilase 4 |
| 2310 | Ciclev10032030m|PACid:20804005 | 43454 | 225 | 30.4 | 10 | 10 | 7 | 7 | 1.531 | 1.181 | 1.151 | AT4G08790.1 | Nitrilase/cyanide hydratase and apolipoprotein N-acyltransferase family protein |
| 1319 | Ciclev10012332m|PACid:20799084 | 37161 | 277 | 25.3 | 8 | 8 | 5 | 5 | 1.537 | 1.136 | 1.382 | AT5G46800.1 | Mitochondrial substrate carrier family protein |
| 2976 | Ciclev10015803m|PACid:20818801 | 44356 | 208 | 13.8 | 11 | 6 | 4 | 3 | 1.538 | 1.261 | 1.493 | AT2G36290.1 | Alpha/beta-Hydrolases superfamily protein |
| 1792 | Ciclev10012766m|PACid:20796866 | 26357 | 34 | 8.1 | 3 | 3 | 1 | 1 | 1.559 | 1.265 | 1.241 | AT3G11402.2 | Cysteine/Histidine-rich C1 domain family protein |
| 2210 | Ciclev10004554m|PACid:20790938 | 83392 | 141 | 8.4 | 8 | 8 | 4 | 4 | 1.575 | 0.952 | 1.226 | AT3G55760.1 | Unknown protein |
| 311 | Ciclev10009013m|PACid:20793441 | 38611 | 160 | 19.3 | 6 | 6 | 4 | 4 | 1.596 | 1.322 | 1.474 | AT1G19140.1 | Unknown protein |
| 1384 | Ciclev10030120m|PACid:20814415 | 144265 | 387 | 8.5 | 16 | 16 | 7 | 7 | 1.621 | 1.307 | 1.306 | AT4G38350.1 | Patched family protein |
| 2270 | Ciclev10014804m|PACid:20816311 | 69431 | 70 | 6.9 | 2 | 2 | 2 | 2 | 1.628 | 1.613 | 1.291 | AT5G63380.1 | AMP-dependent synthetase and ligase family protein |
| 2732 | Ciclev10014599m|PACid:20818542 | 81314 | 60 | 4.8 | 2 | 2 | 2 | 2 | 1.628 | 1.226 | 1.264 | AT1G15060.1 | Uncharacterised conserved protein UCP031088, alpha/beta hydrolase |
| 2848 | Ciclev10028909m|PACid:20813058 | 38377 | 248 | 15.6 | 13 | 13 | 4 | 4 | 1.659 | 1.119 | 1.139 | AT5G64260.1 | EXORDIUM like 2 |
| 1317 | Ciclev10015816m|PACid:20817196 | 45316 | 110 | 11 | 3 | 3 | 2 | 2 | 1.67 | 1.269 | 1.291 | AT4G22930.1 | Pyrimidin 4 |
| 869 | Ciclev10009911m|PACid:20794845 | 17275 | 75 | 8 | 2 | 2 | 1 | 1 | 1.673 | 1.33 | 1.167 | - | Unknown protein |
| 905 | Ciclev10016173m|PACid:20817069 | 35063 | 300 | 31.4 | 11 | 11 | 6 | 6 | 1.717 | 1.415 | 1.576 | AT2G34470.2 | Urease accessory protein G |
| 1238 | Ciclev10009212m|PACid:20794331 | 37689 | 448 | 38.3 | 18 | 8 | 7 | 3 | 1.725 | 1.246 | 1.457 | AT1G12210.1 | RPS5-like 1 |
| 2043 | Ciclev10018836m|PACid:20812059 | 111794 | 141 | 7.6 | 7 | 7 | 6 | 6 | 1.729 | 1.075 | 1.17 | AT4G03200.1 | Catalytics |
| 1732 | Ciclev10014503m|PACid:20816304 | 84522 | 134 | 9.1 | 5 | 5 | 4 | 4 | 1.78 | 1.674 | 1.482 | AT5G20990.1 | Molybdopterin biosynthesis CNX1 protein / molybdenum cofactor biosynthesis enzyme CNX1 (CNX1) |
| 1734 | Ciclev10033542m|PACid:20804403 | 9445 | 106 | 30.6 | 3 | 3 | 2 | 2 | 1.79 | 1.639 | 2.314 | AT5G16660.2 | Unknown protein |
| 2773 | Ciclev10014006m|PACid:20819231 | 458797 | 38 | 0.8 | 2 | 2 | 2 | 2 | 1.794 | 0.9 | 1.081 | AT5G40450.1 | Unknown protein |
| 826 | Ciclev10019303m|PACid:20809121 | 80065 | 77 | 4.3 | 3 | 3 | 2 | 2 | 1.818 | 1.613 | 1.086 | AT4G17620.1 | Glycine-rich protein |
| 3092 | Ciclev10025185m|PACid:20801529 | 79942 | 63 | 4.9 | 2 | 2 | 2 | 2 | 1.834 | 1.285 | 1.078 | AT3G16170.1 | AMP-dependent synthetase and ligase family protein |
| 1601 | Ciclev10010078m|PACid:20794004 | 9114 | 73 | 43.6 | 5 | 5 | 3 | 3 | 1.835 | 1.021 | 1.07 | AT2G06050.1 | Oxophytodienoate-reductase 3 |
| 1283 | Ciclev10007423m|PACid:20793523 | 110696 | 134 | 3.2 | 3 | 3 | 2 | 2 | 1.914 | 1.721 | 2.106 | AT5G57590.1 | Adenosylmethionine-8-amino-7-oxononanoate transaminases |
| 3203 | Ciclev10023691m|PACid:20808690 | 42092 | 267 | 23.4 | 8 | 4 | 5 | 2 | 1.944 | 1.735 | 1.338 | AT4G02340.1 | Alpha/beta-Hydrolases superfamily protein |
| 1829 | Ciclev10007044m|PACid:20790529 | 33904 | 234 | 29.2 | 8 | 8 | 5 | 5 | 2.139 | 1.449 | 1.867 | AT4G35220.1 | Cyclase family protein |
| 491 | Ciclev10022655m|PACid:20807500 | 21350 | 132 | 33.5 | 8 | 8 | 4 | 4 | 2.147 | 1.488 | 1.667 | - | Unknown protein |
| 2854 | Ciclev10022174m|PACid:20810231 | 27146 | 104 | 13.8 | 7 | 7 | 3 | 3 | 2.532 | 1.133 | 1.329 | AT1G17100.1 | SOUL heme-binding family protein |
| 893 | Ciclev10025807m|PACid:20800385 | 50591 | 106 | 5.6 | 4 | 4 | 2 | 2 | 2.619 | 0.858 | 1.725 | AT5G13930.1 | Chalcone and stilbene synthase family protein |
| 877 | Ciclev10021291m|PACid:20808724 | 36239 | 512 | 27.8 | 13 | 13 | 6 | 6 | 2.627 | 1.487 | 1.348 | AT3G62730.1 | Unknown protein |
| 1571 | Ciclev10029240m|PACid:20812600 | 32664 | 146 | 23.4 | 10 | 9 | 5 | 4 | 2.643 | 1.418 | 1.939 | AT2G16060.1 | Hemoglobin 1 |
| 2972 | Ciclev10023108m|PACid:20810325 | 11940 | 302 | 54.1 | 14 | 14 | 4 | 4 | 2.658 | 1.57 | 2.621 | - | Anknown protein |
| 1122 | Ciclev10028838m|PACid:20813945 | 43262 | 141 | 13.9 | 6 | 6 | 4 | 4 | 2.873 | 1.713 | 1.46 | AT3G51000.1 | Alpha/beta-Hydrolases superfamily protein |
| 2105 | Ciclev10001968m|PACid:20785584 | 37352 | 102 | 11.6 | 6 | 4 | 3 | 2 | 3.653 | 1.059 | 2.324 | AT5G64260.1 | EXORDIUM like 2 |
| 2046 | Ciclev10025245m|PACid:20802160 | 75861 | 173 | 7.4 | 5 | 5 | 3 | 3 | 4.543 | 1.511 | 2.815 | AT1G23010.1 | Cu-predoxin superfamily protein |

Note: *Fold change is the ratio of different treatments compared to 0P (the protein abundance under 0P was set as 1). Protein names in bold font were selected to conduct* *qRT-PCR and whose expression levels in different samples were presented in Figure* 7.
